# Clickable bisreactive small gold nanoclusters for preparing multifunctionalized nanomaterials: application to photouncaging of an anticancer molecule†

**DOI:** 10.1039/d3sc04365g

**Published:** 2023-12-15

**Authors:** Kenji Watanabe, Qiyue Mao, Zhouen Zhang, Machi Hata, Masahito Kodera, Hiroaki Kitagishi, Takashi Niwa, Takamitsu Hosoya

**Affiliations:** a Laboratory for Chemical Biology, RIKEN Center for Biosystems Dynamics Research Kobe 650-0047 Japan kenji.watanabe.vs@riken.jp; b Department of Molecular Chemistry and Biochemistry, Faculty of Science and Engineering, Doshisha University Kyotanabe Kyoto 610-0321 Japan; c Laboratory for Molecular Transformation Chemistry, Graduate School of Pharmaceutical Sciences, Kyushu University Higashi-ku Fukuoka 812-8582 Japan; d Laboratory of Chemical Bioscience, Institute of Biomaterials and Bioengineering, Tokyo Medical and Dental University (TMDU) Chiyoda-ku Tokyo 101-0062 Japan thosoya.cb@tmd.ac.jp

## Abstract

In this study, we successfully synthesized a small-sized gold nanocluster (2 nm) coated with homogeneous tripeptides bearing azido and amino groups that enable facile multifunctionalizations. Using sodium phenoxide to reduce tetrachloroauric(iii) acid in the presence of the cysteine-containing tripeptide, we efficiently prepared the gold nanoclusters without damaging the azido group. We then utilized this clickable bisreactive nanocluster as a versatile platform for synthesizing multifunctionalized gold nanomaterials. The resulting nanoclusters were conjugated with an anticancer compound connected to an indolizine moiety for photoinduced uncaging, a photodynamic therapy agent acting as a photosensitizer for uncaging, and a cyclic RGD peptide. The cytotoxicity of the multifunctionalized gold nanoclusters was demonstrated through red light irradiation of human lung cancer-derived A549 cells treated with the synthesized nanomaterials. The significant cytotoxicity exhibited by the cells underscores the potential utility of this method in advanced cancer therapies.

## Abstract 2

2 nm sized gold nanoclusters with azido and amino groups were synthesized. The gold nanoclusters were conjugated with a caged anticancer compound and a photosensitizer for photouncaging, resulting in photoinduced toxicity in cancer cells.

## Graphical abstract

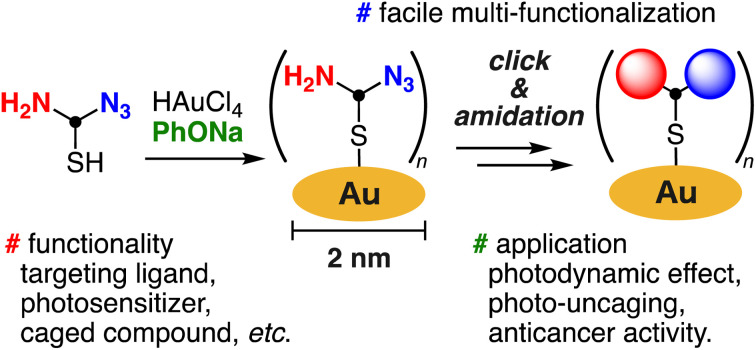

## Introduction

Metal nanoparticles have emerged as a promising class of nanomaterials for biomedical applications, particularly in bioimaging, drug delivery, and photodynamic therapy (PDT).^1,2^ Among the various types of metal nanoparticles, small-sized gold nanoclusters (∼2 nm) have garnered particular interest owing to their unique physical and chemical properties, such as high chemical and photostability.^3–5^ Moreover, these nanoclusters exhibit moderate excretion from the body because of their small size, which provides a good balance between their accumulation at pathological targets and biocompatibility.^6,7^ Their simple synthesis using thiols bearing connecting groups such as azido, amino, and carboxy groups has led to extensive research into developing mono-functionalized gold nanoparticles, such as those carrying drugs, fluorescent dyes, proteins, and DNAs.^8–12^

**Fig. 1:**
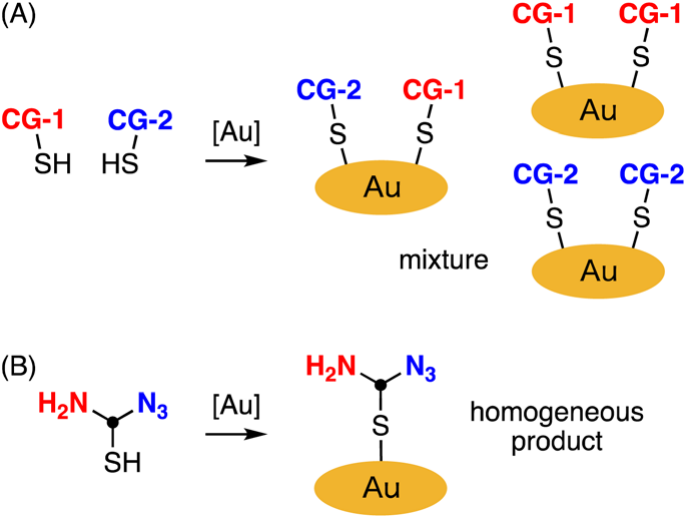
Preparation of gold nanoclucters bearing different connecting groups (CGs). (A) Prepared by random method. (B) Prepared by modification of gold nanoclusters with homogeneous thiol unit containing equivalent amount of azido and amino groups. [Au] = gold source.

As the importance of multimodality in precision medicine grows, the development of nanomaterials with multiple functions has become a subject of significant interest.^13^ Metal nanoparticles with two or more different connecting groups (CGs) serve as valuable intermediates for synthesizing multifunctionalized nanomaterials.^14,15^ However, this approach poses challenges in achieving uniform synthesis of gold nanoclusters since CGs are randomly introduced in various positions and quantities (Fig. 1A).

Considering the crucial role of material homogeneity in ensuring their quality, especially for medical applications, there is a pressing need for a practical method that allows for the controlled introduction of multiple functionalities into gold nanoparticles. We propose that gold nanoclusters homogeneously modified with thiol units containing an equivalent amount of azido and amino groups, suitable for click and amidation reactions, would be instrumental in preparing multifunctionalized nanomaterials (Fig. 1B). While several syntheses of gold nanoclusters containing azido groups have been reported,^9–11^ the incorporation of azido groups has proven challenging due to their instability under typical synthesis conditions. In particular, when attempting to reduce tetrachloroauric(iii) acid (HAuCl_4_) with sodium borohydride (NaBH_4_) in the presence of thiol ligands to obtain gold nanoclusters, a significant portion of the azido groups is reduced to amino groups due to the strong Lewis acidity of HAuCl_4_.^9^ To address this issue, Epple *et al.* reported an excellent method for quantitatively azidating gold nanoclusters functionalized with amines using a diazo transfer reagent.^9^ However, achieving homogeneity requires converting all amino groups to azido groups, making it difficult to prepare equal and uniform amounts of azido and amino groups using this approach. Herein, we present a preparation method for gold nanoclusters modified with homogeneous peptides containing an equivalent amount of azido and amino groups, achieved through optimization of the reductant used during the nanocluster synthesis (Fig. 1B). We also demonstrate the application of this bisreactive gold nanocluster in preparing an anticancer nanomaterial by facilely introducing multiple functionalities *via* click and amidation reactions.

## Results and discussion

### Synthesis of bisreactive gold nanoclusters

**Scheme 1:**
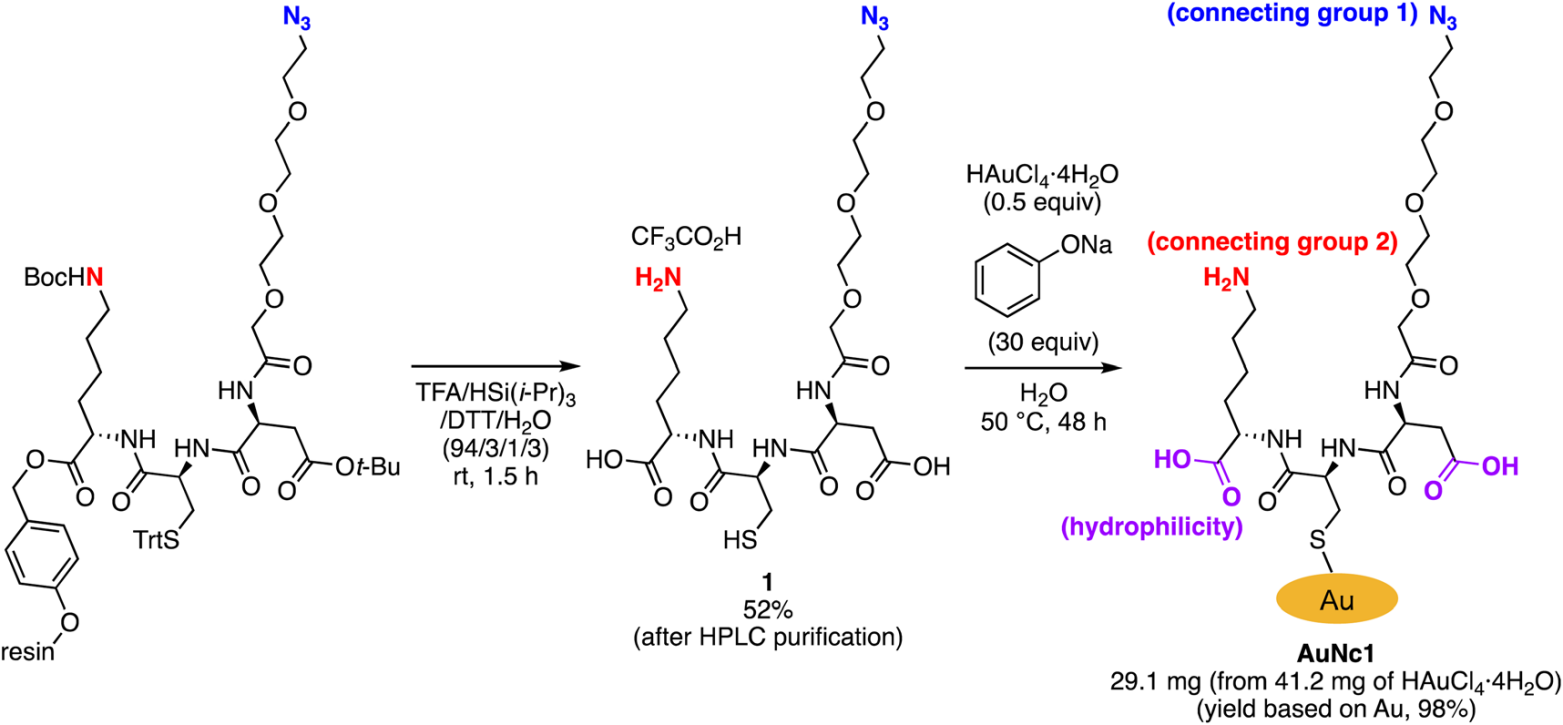
Synthesis of tripeptide thiol 1 and bisreactive AuNc1.

**Fig. 2:**
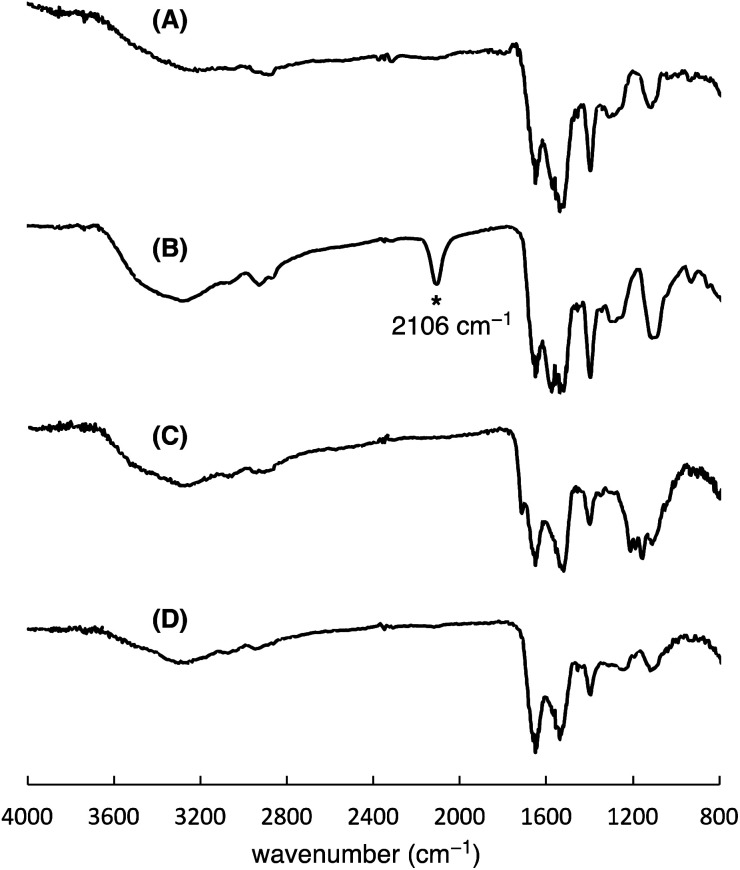
IR spectra of AuNc1 synthesized by using NaBH_4_ (A) or NaOPh (B), and spectra of AuNc2 (C) and AuNc3 (D). The spectra A indicated the absence of the azido groups in AuNc1 probably due to the reduction by NaBH_4_.

**Fig. 3:**
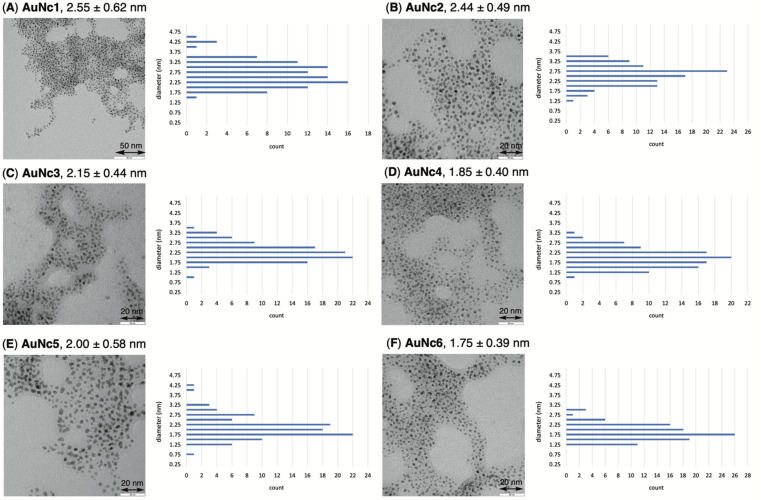
TEM images and particle size distribution data of AuNc1 (A), AuNc2 (B), AuNc3 (C), AuNc4 (D), AuNc5 (E), and AuNc6 (F). Average of 100 counts of particle diameter is shown. The error indicates standard deviation.

To precisely synthesize a bisreactive gold nanocluster, we designed tripeptide thiol 1 with a primary amino group and an azido group (Scheme 1). To enhance hydrophilicity, we incorporated a triethylene glycol spacer and two carboxy groups to the peptide scaffold. The synthesis began with preparing the tripeptide precursor on resin using a solid-phase method (Scheme 1). By employing Diederichsen's conditions with dithiothreitol (DTT) as a trityl group (Trt)-trapping agent,^16^ we smoothly removed the resin and globally deprotected the precursor, yielding the desired tripeptide 1 in moderate yield after purification with high-performance liquid chromatography.^17^ Next, we aimed to prepare the gold nanocluster AuNc1 using a typical method involving the treatment of HAuCl_4_ with tripeptide thiol 1 in the presence of reductants like NaBH_4_ (Table S1†).^18^ However, infrared spectroscopy (IR) analysis indicated the loss of the azido group under these conditions, likely due to its reduction to an amino group, consistent with a previous report^9^ (Fig. 2A, Table S1†). When using the conventional heating condition commonly employed in the synthesis of glutathione-protected gold nanoclusters, no nanoclusters were formed (Table S1†).^19^ To address the vulnerability of the azido group, we explored a synthetic method involving gold nanoclusters covered with tyrosine-containing proteins and peptides.^20,21^ In this approach, the phenoxide group of the tyrosine residue acts as a reducing agent for HAuCl_4_ under basic conditions. Since tripeptide 1 lacks tyrosine residues, we introduced sodium phenoxide to the reaction mixture, successfully leading to the formation of AuNc1 (Scheme 1). Transmission electron microscopy (TEM) measurements revealed that the core size of AuNc1 was approximately 2.55 ± 0.62 nm in diameter, ruling out the presence of larger-sized gold nanoparticles (>5 nm) (Fig. 3A). Notably, the absorption spectrum of AuNc1 did not display the characteristic surface plasmonic resonance band observed in larger-sized gold nanoparticles (Fig. S1†).^22^ The ^1^H NMR analysis of AuNc1 exhibited broadened signals, likely due to the proximity of the peptide moiety to the gold core (Fig. S2A†).^9^ The presence of azido groups was confirmed by the IR absorption spectra at 2106 cm^−1^, confirming the compatibility of the nanocluster forming method with organoazides (Fig. 2B). AuNc1 contained approximately 541 nmol mg^−1^ (31.3 wt%) of ligand 1 as determined by comparing the ^1^H NMR peak integration of the aliphatic hydrogen of ligand 1 in AuNc1 with an internal standard (Fig. S2B†). Inductively coupled plasma atomic emission spectroscopy (ICP-AES) analysis showed that AuNc1 contained 66.3 wt% of gold (Table S2†). Based on the particle size of AuNc1 obtained by the TEM measurement, we assumed that a single AuNc1 particle contained about 333 gold atoms.^23^ Base on these results, the number of ligand 1 in a single AuNc1 particle was estimated to be approximately 54. The ligand 1 with relatively larger molecular size (molecular weight = 578.6) was found to be introduced at lower densities on the gold nanoclusters compared to the one covered with azidated glutathione (MW = 332.3) reported by Epple *et al.*^9^

### Functionalization and characterization of gold nanoclusters

**Scheme 2:**
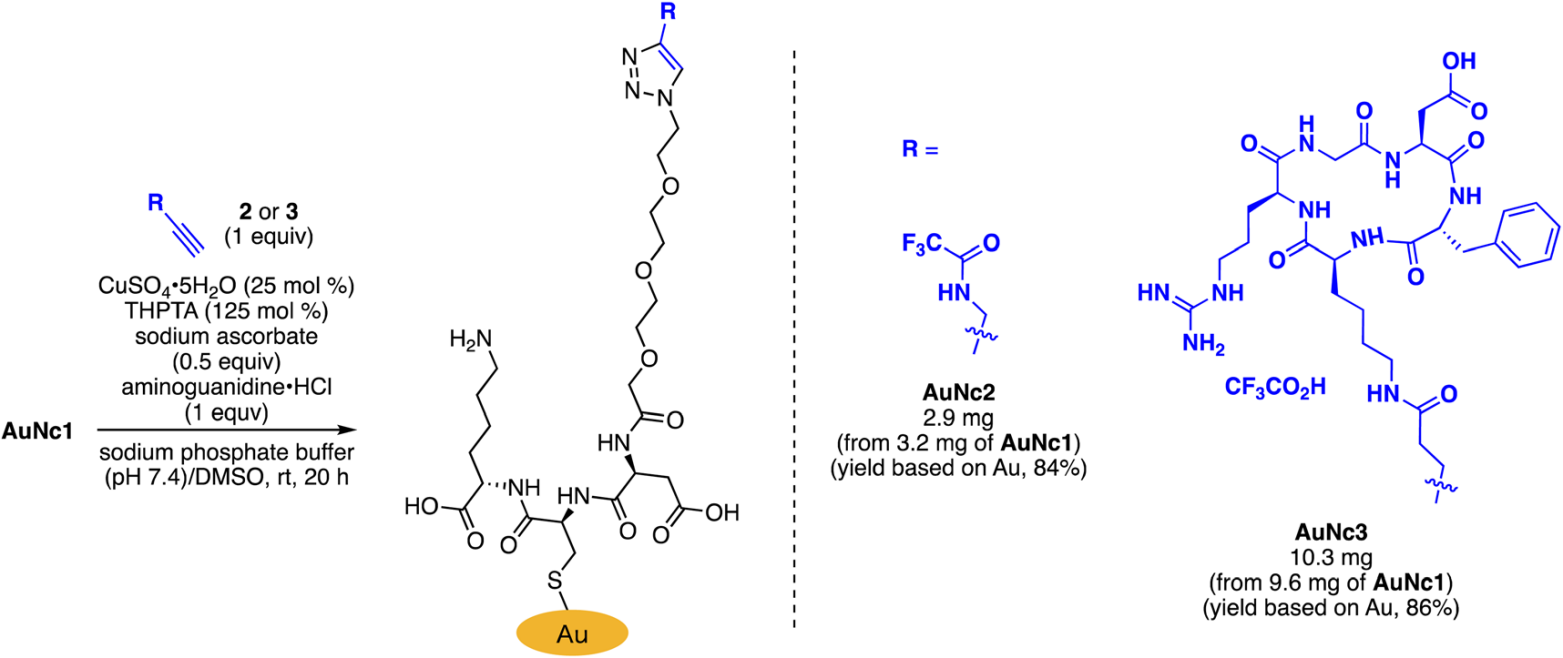
Derivatization of AuNc1 by CuAAC.

To assess the quantity of introduced azido groups, we conducted a copper-catalyzed azide–alkyne cycloaddition (CuAAC)^9,11,24,25^ between AuNc1 and *N*-propargyltrifluoro-acetamide (2), resulting in AuNc2 containing CF_3_ groups (Scheme 2, Fig. 3B). The disappearance of the azido peak (2106 cm^−1^) in the IR analysis of AuNc2 indicated that most of the azido groups of AuNc1 were consumed (Fig. 2C). ^1^H and ^19^F NMR analysis indicated AuNc2 contained 287 nmol mg^−1^ of CF_3_ groups and 299 nmol mg^−1^ of ligands (Fig. S3†). These results indicated that the azido groups were converted in a nearly quantitative manner (96% efficiency, Fig. S3†). The number of ligands in a single AuNc2 particle was estimated to be approximately 32.^26^ Note that AuNc1 was also applicable to a strain-promoted azide–alkyne cycloaddition (SPAAC) reaction,^10,27–29^ affording the product efficiently (Fig. S4†).

**Scheme 3:**
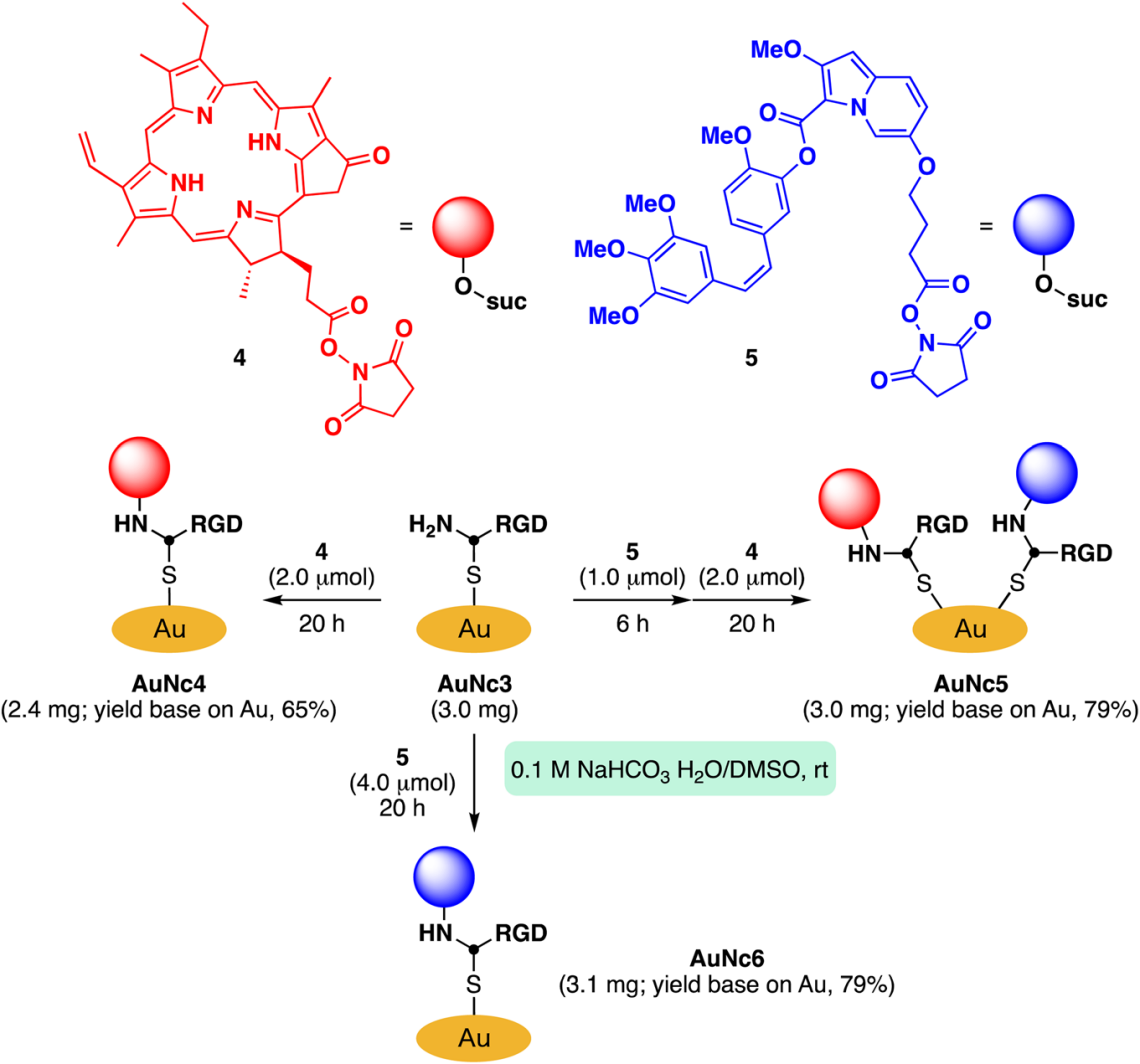
Further derivatization of AuNc3 with a photosensitizer and a caged compound.

To demonstrate the practicality of bisreactive AuNc1 in introducing multiple functionalities, we explored the introduction of anticancer materials to create a potential nanomedicine candidate. First, we focused on a cyclic RGD peptide, known for its specific binding to integrin α_v_β_3_, which is abundantly expressed in cancer cells.^30^ We conjugated the cyclic RGD peptide to deliver the gold nanoclusters into the cancer cells.^31^ The CuAAC reaction of AuNc1 with RGD-derived alkyne 3 (ref. 30) smoothly led to the formation of AuNc3, as characterized by TEM analysis (Scheme 2, Fig. 3C). The disappearance of the IR absorbance corresponding to the azido group suggested that AuNc3 was fully decorated with 3 (Fig. 2D). Next, we utilized the remaining amino groups to link pyropheophorbide a, a potent photosensitizer (PS) used in PDT.^32,33^ The mixing of the *N*-hydroxysuccinimidyl (NHS) ester derivative 4 with AuNc3 resulted in PS-conjugated AuNc4, expected to function not only as a PDT agent but also as a photosensitizer to trigger photouncaging (*vide infra*) (Scheme 3, Fig. 3D, S5A†). The absorption intensity at 678 nm of AuNc4 indicated the presence of 209 nmol mg^−1^ of pyropheophorbide a moiety, corresponding to approximately 24 units incorporated on average on a single AuNc4 particle.^26^

### Synthesis of gold nanocluster-based caged material

We proceeded to utilize the bisreactive AuNc1 as a platform for creating an anticancer agent-caged material. Caged compounds are precursor molecules protected with a photocleavable group, which can release the original bioactive molecule upon light irradiation.^34^ In recent times, several research groups have reported caged compounds that respond to low-energy long-wavelength light.^35–40^ We have also developed an uncaging system based on the photooxidation of indolizine cores (Scheme S1†).^41–43^ This system allows for a rapid release of caged molecules with the help of singlet oxygen generated by biopermeable red light irradiation in the presence of a separately added PS. This enables efficient uncaging of carboxylic acids or alcohols. We demonstrated that this system effectively uncages anticancer drugs under a hypoxic environment.^41^ However, applying this method to cell or animal experiments posed challenges due to a significant decrease in the reaction rate, likely caused by the diffusion of separately added indolizine cores and PS. To tackle this issue, we hypothesized that gold nanoclusters would provide a useful platform for accumulating functional moieties, including our uncaging system.

Based on this concept, we synthesized indolizine NHS ester 5 to cage the anticancer drug combretastatin A4 (CA4) (Scheme 3). Treating RGD-conjugated AuNc3 with NHS ester 5 for a short time (6 h) partially filled the amine moieties. Subsequent addition of an excess amount of PS-containing NHS ester 4 fully covered the remaining amino groups, resulting in the trifunctionalized AuNc5 (Scheme 3, Fig. 3E, S5B†).^44^ The absorbance at 678 nm confirmed that AuNc5 contained 143 nmol mg^−1^ of pyropheophorbide a. We estimated the quantity of indolizine–CA4 moiety through an uncaging reaction under red light irradiation (660 nm) in phosphate-buffered saline (PBS (−)). By fitting the data with a pseudo-first order rate equation, we calculated that the maximum amount of CA4 released from AuNc5 was 80.4 nmol mg^−1^ (Fig. S6†). Thus, the ratio and total amount of pyropheophorbide a and CA4 on AuNc5 were determined to be approximately 1.8 : 1 and 223 nmol mg^−1^, respectively. As a control material, we prepared AuNc6 without a PS moiety using AuNc3 and 5, and confirmed that CA4 was not released from AuNc6 (see the ESI Section S8†). This result emphasizes the essential role of pyropheophorbide a in the photoreaction of indolizines.

The size histogram of the AuNc3–6 in TEM measurements showed a decrease in the 3–4 nm-sized particles and a convergence to a group of 2 nm-sized particles (Fig. 3). Such transition in particle size by partial decomposition of gold nanoclusters has been reported previously.^45^

### Evaluation of anticancer activity

**Fig. 4:**
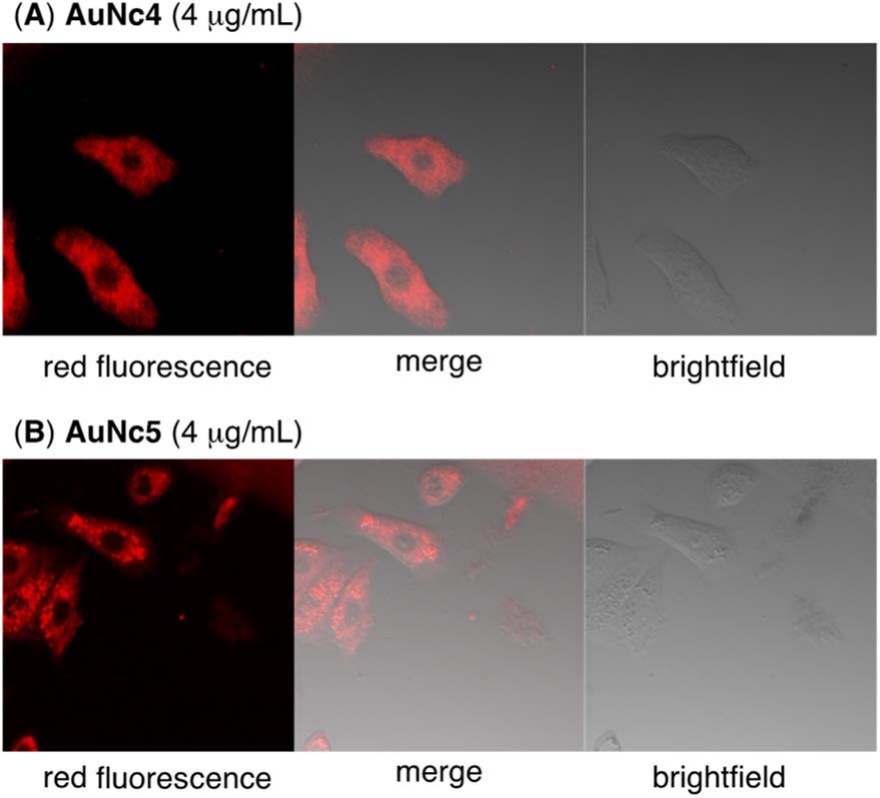
Confocal fluorescence microscopy imaging of A549 cells incubated with AuNc4 (A, 4 μg mL^−1^) or AuNc5 (B, 4 μg mL^−1^) for 1 h in D-10 medium. The excitation and fluorescence wavelength were 638 nm and 688 nm, respectively.

**Fig. 5:**
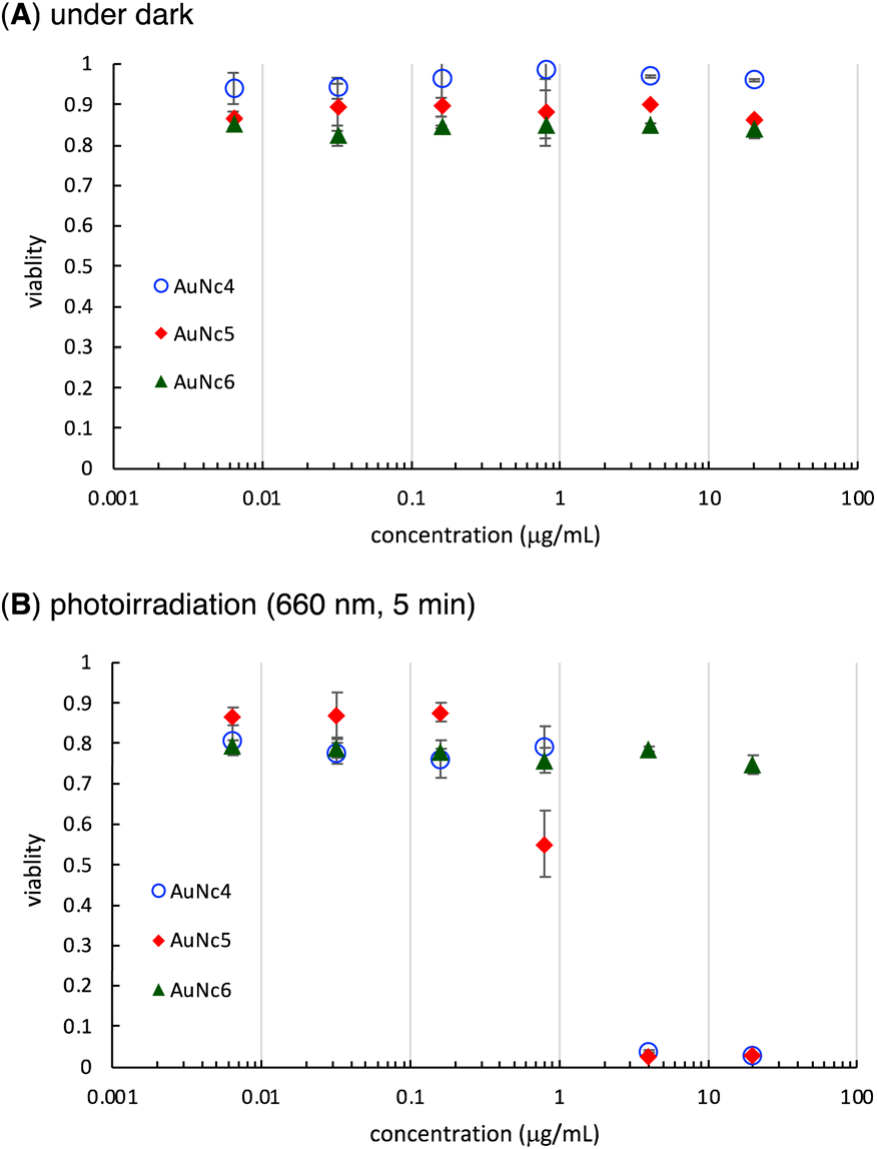
Cytotoxicity of AuNc4–6 on A549 cells under dark (A) and photoirradiation conditions (B). Averages and standard deviations of three independent experiments are shown.

Finally, we evaluated the photoinduced cytotoxicity of the multifunctionalized gold nanoclusters using A549 cells derived from human lung cancer, which express high levels of integrin α_v_β_3_ and are sensitive to CA4.^46^ The methyl thiazolyl tetrazolium assay was used for cellular experiments. A549 cells were cultured in Dulbecco's modified eagle medium supplemented with 10% fetal bovine serum albumin (D-10) containing gold nanoclusters for 1 h. Subsequently, the medium was replaced with fresh D-10 to eliminate the effect of gold nanoclusters that were not incorporated into the cells before photoirradiation. Fluorescence of pyropheophorbide a was observed using confocal microscope imaging to confirm the uptake of AuNc4 and AuNc5 in A549 cells (Fig. 4). In control experiments conducted in the dark without photoirradiation, the gold nanoclusters AuNc4–6 exhibited almost no toxicity (Fig. 5A). However, under photoirradiation with red light at 660 nm for 5 min, AuNc4 and AuNc5 demonstrated concentration-dependent photocytotoxicity, while non-photoreactive AuNc6 showed negligible toxicity (Fig. 5B). The phototoxicity of AuNc4 is attributed to the PDT effect of pyropheophorbide a.^32^ Notably, AuNc5 displayed increased cytotoxicity, indicating that the release of CA4 enhanced cell death in conjunction with the PDT effect (Fig. 5B). Unfortunately, AuNc4 and AuNc5 also exhibited substantial photoinduced toxicity to the normal cells derived from human lung fibroblasts (WI-38)^47^ under the photoirradiation conditions (Fig. S7†). The interaction of the RGD moieties of AuNc4 and AuNc5 with α_v_β_3_ receptors on WI-38 cells could lead to the toxicity.^48^ These results highlight the importance of selective photoirradiation of the pathological tissues for the safe and effective application of this approach in cancer treatment.

## Conclusions

We successfully synthesized a clickable small (2 nm) gold nanocluster covered with homogeneous tripeptides containing azido and amino groups, facilitating the easy introduction of multiple functionalities. The key to this successful preparation was the use of sodium phenoxide as a mild reductant of HAuCl_4_ in the presence of the tripeptide, leaving the azido groups untouched. By employing this bisreactive nanocluster, we created a novel anticancer material, connecting a cyclic RGD peptide, a photoreactive indolizine–anticancer agent conjugate, and a PS through click and amidation reactions, and assessed their photoinduced cytotoxic activities using A549 cells. Our findings suggest that the multifunctionalizable gold nanocluster platform is valuable for constructing carriers for PDT and caged materials. Furthermore, our approach utilizing homogeneous peptide modification may unlock new possibilities for developing various multifunctionalized nanomaterials for biomedical applications. To enhance their utility, we are currently working on developing uniform tri- or more multiply functionalized nanoclusters.

## Data availability

All the data supporting this article have been included in ESI.†

## Author contributions

Methodology, KW, QM, MH; supervision, ZZ, MK, HK, TN, TH; funding acquisition, KW, HK, TN, TH; writing, KW, TN, TH. All authors discussed and commented on the manuscript.

## Conflicts of interest

The authors declare no conflicts of interest.

## Supplementary Material

SC-015-D3SC04365G-s001
