# Supplementary material for: Clickable bisreactive small gold nanoclusters for preparing multifunctionalized nanomaterials: application to photouncaging of an anticancer molecule
Source: Chem Sci. 2023 Dec 15;15(4):1402–8. doi: 10.1039/d3sc04365g (PMC10806826; doi:10.1039/d3sc04365g)

Electronic Supplementary Material (ESI) for Chemical Science.
This journal is © The Royal Society of Chemistry 2023

Electronic Supplementary Information (ESI) for

Clickable bisreactive small gold nanoclusters for preparing multifunctionalized nanomaterials: application to photouncaging of an anticancer molecule

Kenji Watanabe,*[a] Qiyue Mao,[b] Zhouen Zhang,[a] Machi Hata,[b] Masahito Kodera,[b] Hiroaki Kitagishi,[b] Takashi Niwa,[acd] and Takamitsu Hosoya*[ad]

[a]Laboratory for Chemical Biology, RIKEN Center for Biosystems Dynamics Research, Kobe 650-0047, Japan
[b]Department of Molecular Chemistry and Biochemistry, Faculty of Science and Engineering, Doshisha University, Kyotanabe, Kyoto 610-0321, Japan
[c]Laboratory for Molecular Transformation Chemistry, Graduate School of Pharmaceutical Sciences, Kyushu University, Higashi-ku, Fukuoka 812-8582, Japan
[d]Laboratory of Chemical Bioscience, Institute of Biomaterials and Bioengineering, Tokyo Medical and Dental University, Chiyoda-ku, Tokyo 101-0062, Japan

Table of contents

1. Unsuccessful synthesis of **AuNc1** (Table S1) (S2–S3)
2. Absorption spectrum of **AuNc1** (Fig. S1) (S4)
3. ^{1}H NMR spectra of **AuNc1** (Fig. S2) (S5)
4. ^{19}F and ^{1}H NMR spectra of **AuNc2** (Fig. S3) (S6)
5. SPAAC of **AuNc1** (Fig. S4) (S7–S8)
6. IR spectra of **AuNc4–6** (Fig. S5) (S9)
7. Red-light induced uncaging system based on photooxidation of Indolizines (**Scheme S1**) (S10)
8. Quantification of CA4 released from **AuNc5** (Fig. S6) (S11)
9. Cytotoxicity of **AuNc4–6** on WI-38 cells (Fig. S7) (S12)
10. General information (S13)
11. Chemicals (S14)
12. Synthetic procedures for low-molecular-weight compounds (S15–S18)
13. Synthetic procedures for gold nanoclusters (S19–S21)
14. Procedure for TEM measurements (S22)
15. Procedure for ICP-AES measurements (S23)
16. Procedure for MTT assay (S24)
17. Procedure for confocal fluorescence microscopy imaging (S25)
18. References (S26)
19. NMR charts (S27–S31)

1. Unsuccessful synthesis of AuNc1 (Table S1)

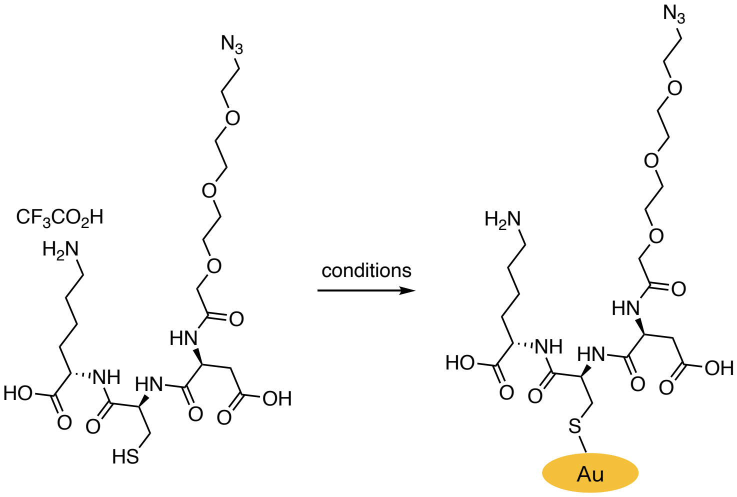

1 AuNc1

entry	conditions	comments
1	$HAuCl_4 \cdot 4H_2O$ (0.2 equiv), TOAB (0.22 equiv), $NaBH_4$ (4 equiv), MeOH/H_2O (1/1), 0 °C to rt, 18 h	azido groups were reduced
2	$HAuCl_4 \cdot 4H_2O$ (0.68 equiv), H_2O, 70 °C, 24 h	nanoclusters were not obtained

TOAB = tetraoctylammonium bromide

Experiment procedure:

(Table S1, entry 1)

To a solution of $HAuCl_4 \cdot 4H_2O$ (1.7 mg, 4.1 µmol, 0.2 equiv) in MeOH (0.15 mL) in a 4 mL vial was added a solution of tetraoctylammonium bromide (TOAB, 2.4 mg, 4.4 µmol, 0.22 equiv) in MeOH (0.15 mL). The solution was stirred at room temperature for 20 min under argon atmosphere. To this solution was added a solution of peptide **1** (13.9 mg, 20.0 µmol, 1 equiv) in MeOH (0.30 mL). The mixture was stirred at 0 °C for 30 min under argon atmosphere. A solution of $NaBH_4$ (3.0 mg, 80 µmol, 4 equiv) in ultrapure water (0.60 mL) was added to the reaction mixture. The mixture was stirred at room temperature for 18 h under argon atmosphere. After the reaction, the reaction mixture was diluted with ultrapure water (12 mL). The solution was filtered through a syringe filter (0.22 µm) and subjected to ultrafiltration using an Amicon Ultra filter unit (NMWL 3000) for 5 cycles. The concentrated solution was freeze-dried to yield a black solid (2.5 mg).

(Table S1, entry 2)

To a solution of peptide **1** (22.2 mg, 32.0 µmol, 1.5 equiv) in ultrapure water (10 mL) in a 20 mL

vial was added a solution of $HAuCl_4 \cdot 4H_2O$ (8.8 mg, 21 μmol, 1.0 equiv) in ultrapure water (0.70 mL). The resulting solution was stirred at 70 °C for 24 h under argon atmosphere. After the reaction, the reaction mixture was subjected to ultrafiltration using an Amicon Ultra filter unit (NMWL 3000) for five cycles. The concentrated solution was freeze-dried. However, almost no compound was obtained, indicating that the gold nanoclusters were not formed.

2. Absorption spectrum of AuNc1 (Fig. S1)

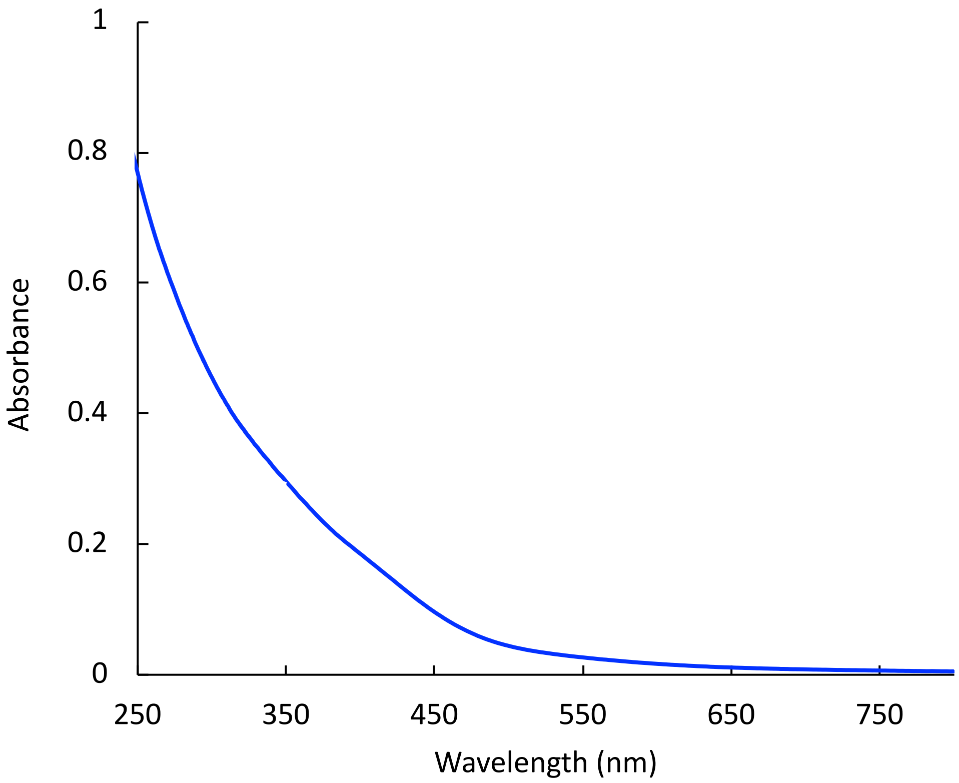

Fig. S1 Absorption spectrum of **AuNc1** (50 μg/mL) in pH 7.4 sodium phosphate buffer (0.1 M). The absorption spectrum of **AuNc1** did not show the surface plasmonic resonance (SPR) band observed in larger-sized gold nanoparticles.

3. ^{1}H NMR spectra of AuNc1 (Fig. S2)

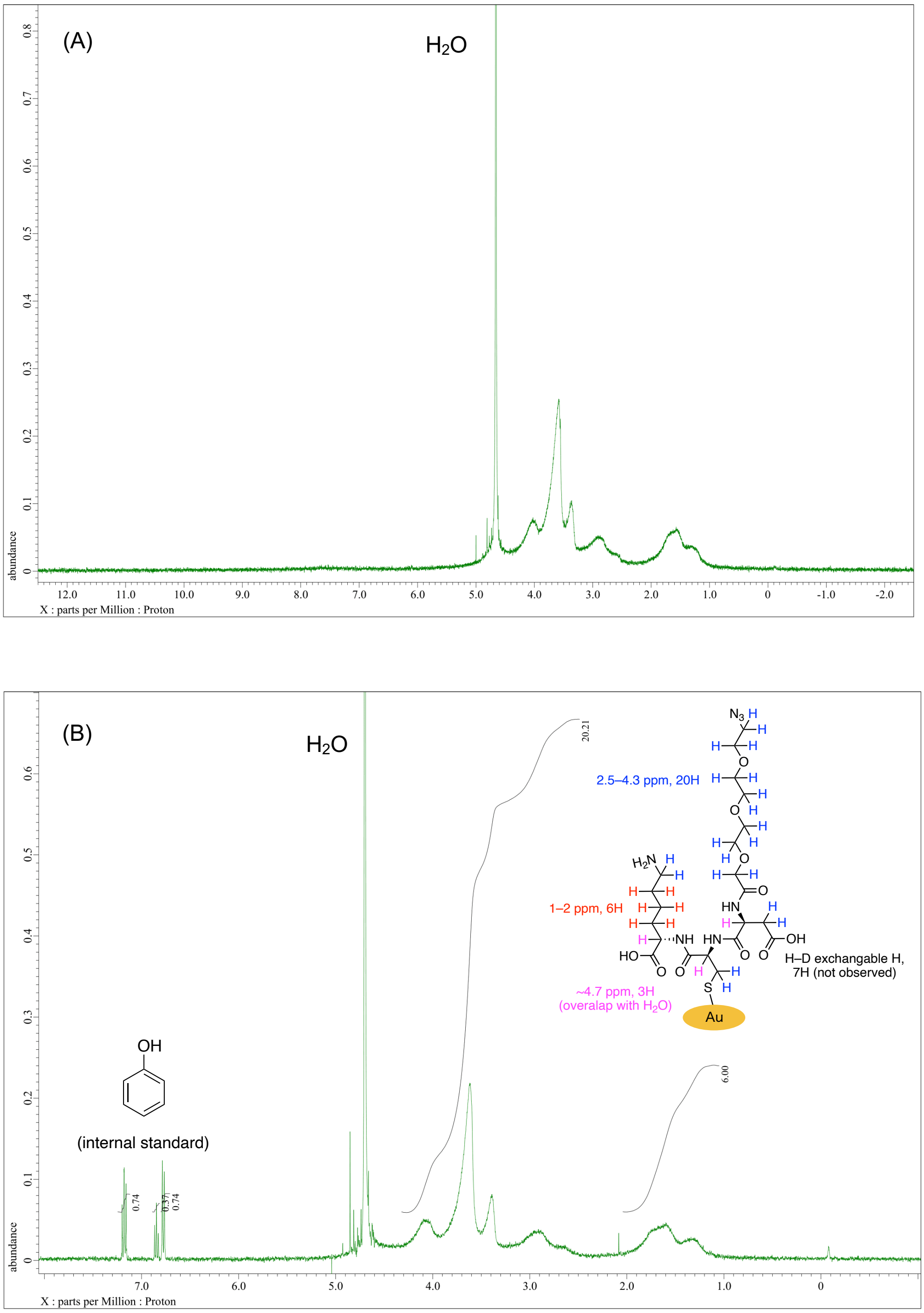

Fig. S2 (A) ^{1}H NMR spectrum of **AuNc1** (6.4 mg/mL) in water-d_2. Broadened signals due to the proximity of the peptide moiety to the gold core were observed. (B) ^{1}H NMR spectrum of **AuNc1** (3.5 mg) in water-d_2 (700 μL) containing phenol (700 nmol) as an internal standard. 1 mg of **AuNc1** contained 541 nmol of tripeptide thiol ligand **1**.

4. ^{19}F and ^{1}H NMR spectra of AuNc2 (Fig. S3)

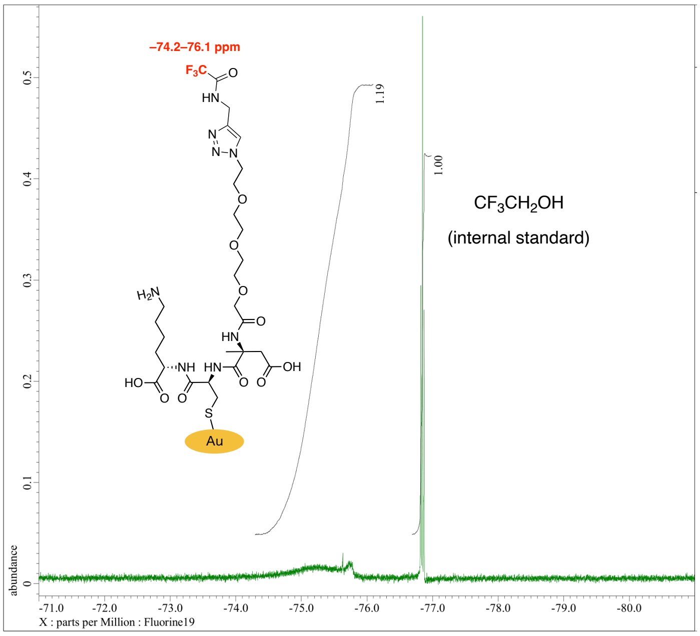

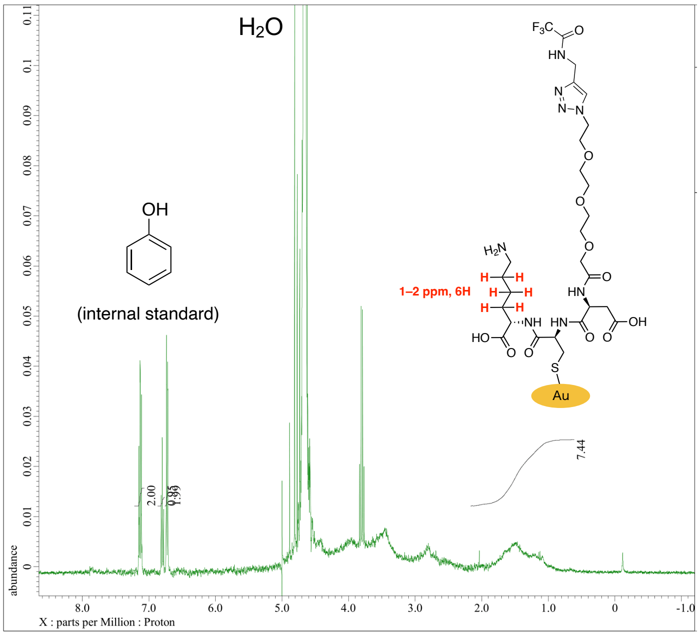

Fig. S3 ^{19}F (A) and ^{1}H (B) NMR spectra of **AuNc2** (2.9 mg) in water-d_2 (700 µL) containing 2,2,2-trifluoroethanol (700 nmol) and phenol (700 nmol) as internal standards. 1 mg of **AuNc2** contained 287 nmol of CF_3 groups and 299 nmol of ligands, respectively. The efficiency of the click reaction was estimated to be 96%.

5. SPAAC of AuNc1 (Fig. S4)

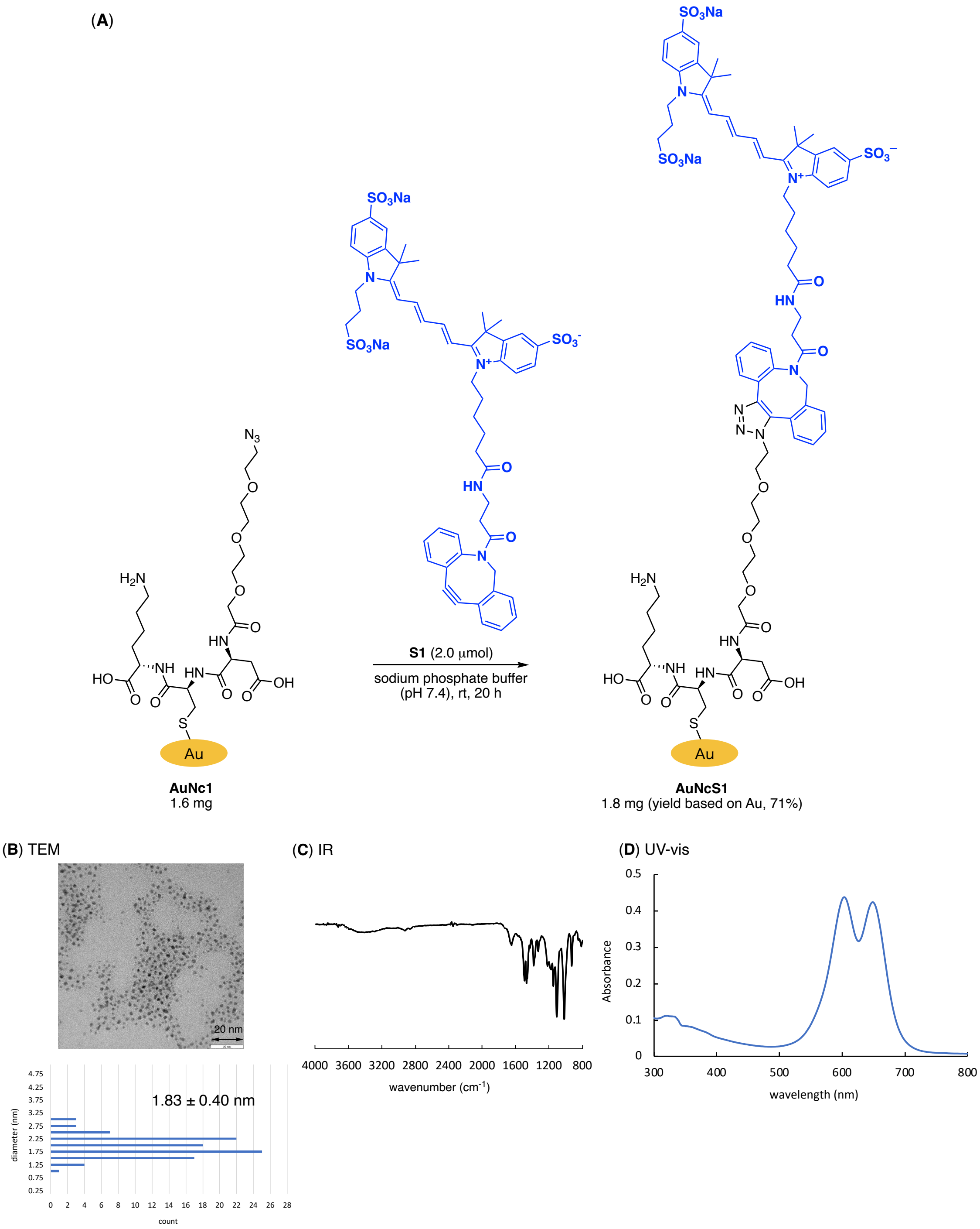

Fig. S4 (A) Derivatization of **AuNc1** with a cyanine dye **S1** via a strain-promoted azide–alkyne cycloaddition (SPAAC) reaction. (B) Transmission electron microscopy (TEM) analysis of the product **AuNcS1** indicated the particle size of 1.83 ± 0.40 nm. (C) IR spectrum of **AuNcS1** indicated clean consumption of the azido groups. (D) UV-vis spectrum of **AuNcS1** (10 μg/mL)

in a sodium phosphate buffer (pH 7.4). The blue shift of the absorption maximum wavelength was observed. The estimated amount of introduced cyanine dyes **S1** was at least 204 nmol/mg based on absorption of **AuNcS1** at 649 nm where the gold nanoclusters themselves did not show absorption. Absorption coefficiency of **S1** (ε_{649} = 214,000 M^{-1} cm^{-1}) was used for the estimation.

Inductively coupled plasma atomic emission spectroscopy (ICP-AES) analysis showed that **AuNcS1** contained 41.8 wt% of gold (Table S2). Based on the particle size of **AuNcS1** obtained by the TEM measurement, a single **AuNc1** particle was assumed to contain approximately 250 gold atoms.[S1] The number of cyanine dyes **S1** in a single **AuNcS1** particle was estimated to be approximately 24.

6. IR spectra of AuNc4–6 (Fig. S5)

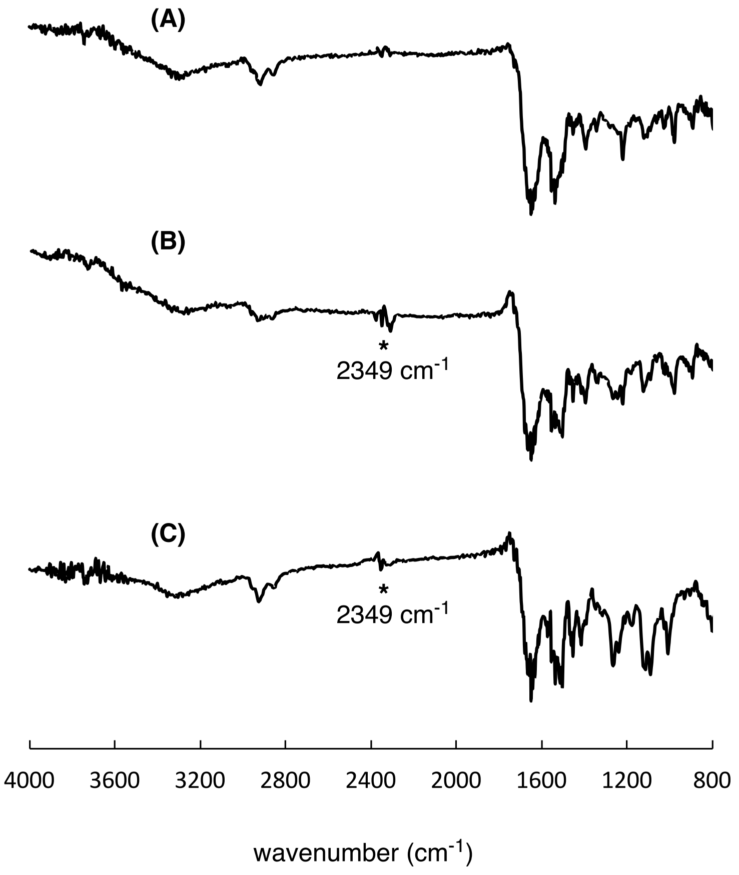

Fig. S5. IR spectra of **AuNc4** (A), **AuNc5** (B), and **AuNc6** (C). The background peak at 2349 cm^{-1} (*) is due to CO_2.

7. Red-light induced uncaging system based on photooxidation of indolizine (**Scheme S1**)

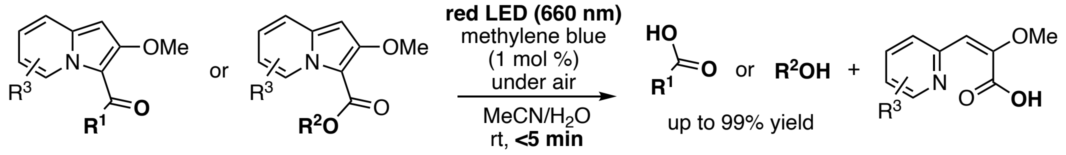

8. Quantification of CA4 released from AuNc5 (Fig. S6)

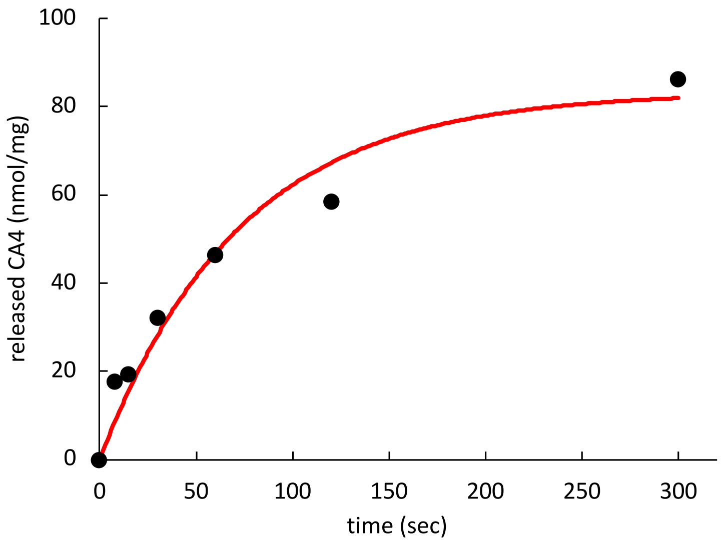

Fig. S6. Representative fitting results. Increase of CA4 from t_0 to t_{max} (n) = 80.4 ± 2.5 (mg/mL) (83.3 (1st), 78.5 (2nd), 79.4 (3rd))

Experiment procedure:

A solution of **AuNc5** or **AuNc6** (0.05 mg/mL, 150 μL) in PBS(–) (pH 7.4, 138 mM NaCl, 2.7 mM KCl, 10 mM phosphate) was added to a 4 mL vial. The vial was placed in an PhotoRedOx box (EvoluChem) equipped with a H160 Tuna Flora LED lamp (Kessil, 660 nm). The reaction mixture was photoirradiated for 0–300 sec at room temperature under air while stirring with a magnetic stirrer. A calibration curve for CA4 was prepared using a Thermo Fisher Scientific DIONEX Ultimate 3000 ultra-high-performance liquid chromatography (UPLC) system coupled with a Q Exactive Orbitrap mass spectrometer (LC/MS/MS), and the amount of CA4 produced in the reaction solution after the photoirradiation was quantified.

The maximum amount of CA4 released from **AuNc5** was calculated by the following pseudo-first-order rate equation (Figure S1):

$$y = n\,(1 - \exp(-\,m * t))$$

where t, y, n and m stand for time, amount of CA4 at t, increase of CA4 from t_0 to t_{max}, apparent rate constant (k_{obs}), respectively.

Three independent experiments were performed for repeatability.

For the experiment using **AuNc6**, almost no release of CA4 was observed.

9. Cytotoxicity of AuNc4–6 on WI-38 cells (Fig. S7)

(**A**) under dark

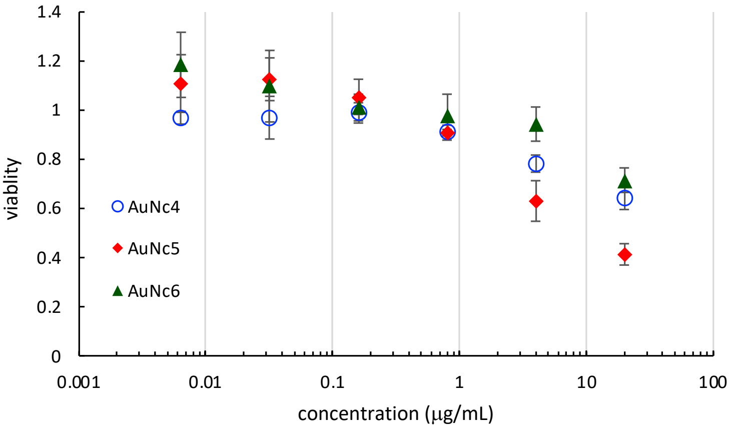

(**B**) photoirradiation (660 nm, 5 min)

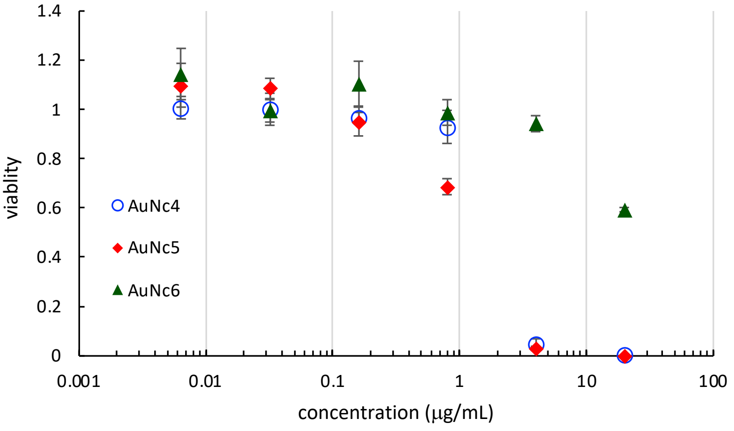

Fig. S7. Cytotoxicity of **AuNc4–6** on WI-38 cells under dark (A) and photoirradiation conditions (B). Averages and standard deviations of three independent experiments are shown.

10. General information

All organic synthesis were performed under argon atmosphere and shading from light unless otherwise indicated. The definition for room temperature (rt) is 25 ± 2 °C. An IKA RCT basic hot plate stirrer equipped with aluminum blocks was used for heating. Analytical thin-layer chromatography (TLC) was performed on precoated (0.25 mm) silica-gel plates (Merck, Merck Silica Gel 60 F254). Column chromatography was conducted on a YAMAZEN Automated Flash Chromatography System that consists of AI-580 and Parallel Frac FR-360. Melting points (mp) were measured with an OptiMelt automated melting point apparatus (Stanford Research Systems, Inc.) and were uncorrected. ^{1}H NMR (400 MHz), ^{13}C NMR (100 MHz), and ^{19}F NMR (373 MHz) spectra were obtained from measurements at room temperature on a JEOL ECS400 spectrometer. Chloroform-d_1 ($CDCl_3$) containing 0.05% tetramethylsilane (TMS, 99.8%D, Cambridge Isotope Laboratories) and water-d_2 (D_2O, Cambridge Isotope Laboratories) were used as solvents for NMR measurements. Chemical shifts (δ) for ^{1}H NMR are given in parts per million (ppm) downfield from signal of TMS (δ 0.00 ppm) and residual water (δ 4.70 ppm) for the measurements in $CDCl_3$ and D_2O, respectively. The residual chloroform (δ 77.2 ppm) was used as an internal standard for the $^{13}C\{^{1}H\}$ NMR measurements in $CDCl_3$. Acetonitirle (δ 119.7 ppm) was used as an external standard for $^{13}C\{^{1}H\}$ NMR measurements in D_2O. Chemical shifts for ^{19}F NMR are given in parts per million downfield from signal of (trifluoromethyl)benzene ($PhCF_3$, δ –62.6 ppm in $CDCl_3$) as external standard. The coupling constants (*J*) are given in hertz (Hz). The abbreviations s, d, t, q, and m signify singlet, doublet, triplet, quartet, and multiplet, respectively. UV-vis spectra were recorded on a Shimazu UV-1800 spectrometer. IR spectra were measured by attenuated total reflection method on a Shimadzu IRPrestige-21 spectrometer with the absorption band given in cm^{-1}. Recycle gel permeation chromatography (GPC) was performed on a YMC LC-Forte/R multiple preparative HPLC system. High-resolution mass spectra (HRMS) were measured on a Thermo Fisher Scientific Exactive Plus Orbitrap mass spectrometer. Liquid chromatography tandem mass spectrometry (LC-MS/MS) was performed on a Thermo Fisher ScientificQ Exactive Orbitrap mass spectrometer equipped with a DIONEX Ultimate 3000 ultra-high-performance liquid chromatography (UPLC) system. High-performance liquid chromatography (HPLC) was performed on Shimadzu HPLC Systems that consists of LC-20AR (liquid chromatograph), DGU-20A5R (degasser), SPD-M20A (diode array detector), CBM-20A (communication bus), and FRC-40 (fraction collector) units.

11. Chemicals

All purchased chemicals were used as received unless otherwise indicated. Diethyl ether, ultrapure water, tetrahydrofuran (THF, deoxygenated), dichloromethane (CH_2Cl_2, deoxygenated) methanol (MeOH, super dehydrated), nitric acid (69 wt%), and hydrochloric acid (35 wt%) were purchase from FUJIFILM Wako Pure Chemical. *N*,*N*-Dimethylformamide (DMF, $H_2O < 50$ ppm), piperidine, *N*,*N*-diisopropylethylamine (EtN(*i*-Pr)$_2$), triisopropylsilane (HSi(*i*-Pr)$_3$), trifluoroacetic acid, DL-dithiothreitol (DTT), acetonitrile (MeCN, HPLC grade), triphosgene, sodium sulfate, ethyl acetate (EtOAc), dimethylsulfoxide (DMSO), formic acid, hydrogen tetrachloroaurate(III) tetrahydrate ($HAuCl_4 \cdot 4H_2O$), sodium hydroxide (NaOH), tetraoctylammonium bromide (TOAB), sodium phosphate buffer (100 mM, pH 7.4), copper(II) sulfate pentahydrate ($CuSO_4 \cdot 5H_2O$), sodium L-ascorbate, ethylenediaminetetraacetic acid (EDTA, 0.5 M, pH 8.0), sodium hydrogen carbonate ($NaHCO_3$), phosphate buffered saline (PBS(–), pH 7.4, 138 mM NaCl, 2.7 mM KCl, 10 mM phosphate), ethanol (EtOH, 95%), and 2,2,2-trifluoroethanol were purchased from Nacalai Tesque. 1-[Bis(dimethylamino)methylene]-1*H*-1,2,3-triazolo[4,5-*b*]pyridinium 3-oxide hexafluorophosphate (HATU), 1-hydroxybenzotriazole (HOAt), 2-(2-(2-(2-azidoethoxy)ethoxy)ethoxy)acetic acid, phenol, *N*,*N*,*N'*,*N'*-tetramethyl-*O*-(*N*-succinimidyl)uronium tetrafluoroborate (TSTU), aminoguanidine hydrochloride sodium borohydride ($NaBH_4$), and *N*-propargyltrifluoroacetamide (**2**) were purchased from Tokyo Chemical Industry. 4-*tert*-Butyl *N*-[(9*H*-fluoren-9-ylmethoxy)carbonyl]-L-aspartate (Fmoc-L-Asp(*t*-Bu)-OH) and tris(3-hydroxypropyltriazolylmethyl)amine (THPTA) were purchase from Merck. Fmoc-L-Lys(Boc)-Wang resin, *N*-(((9H-fluoren-9-yl)methoxy)carbonyl)-*S*-trityl-L-cysteine (Fmoc-L-Cys(trt)-OH), and 1-(3-dimethylaminopropyl)-3-ethylcarbodiimide hydrochloride (EDC·HCl) were purchase from Watanabe Chemical Industries. Pyropheophorbide a was purchased from Frontier Specialty Chemicals. Combretastatin A4 was purchased from BLD Pharmatech. Cyanine dye **S1** was purchased from BroadPharm. A cyclic RGD peptide **3**[S2] and *tert*-butyl 4-((2-methoxyindolizin-6-yl)oxy)butanoate[S3] were synthesized according to the literature.

12. Synthetic procedures for low-molecular-weight compounds

(13*S*,16*R*,19*S*)-1-Azido-19-carboxy-13-(carboxymethyl)-16-(mercaptomethyl)-11,14,17-trioxo-3,6,9-trioxa-12,15,18-triazatricosan-23-aminium trifluoroacetate salt (**1**)

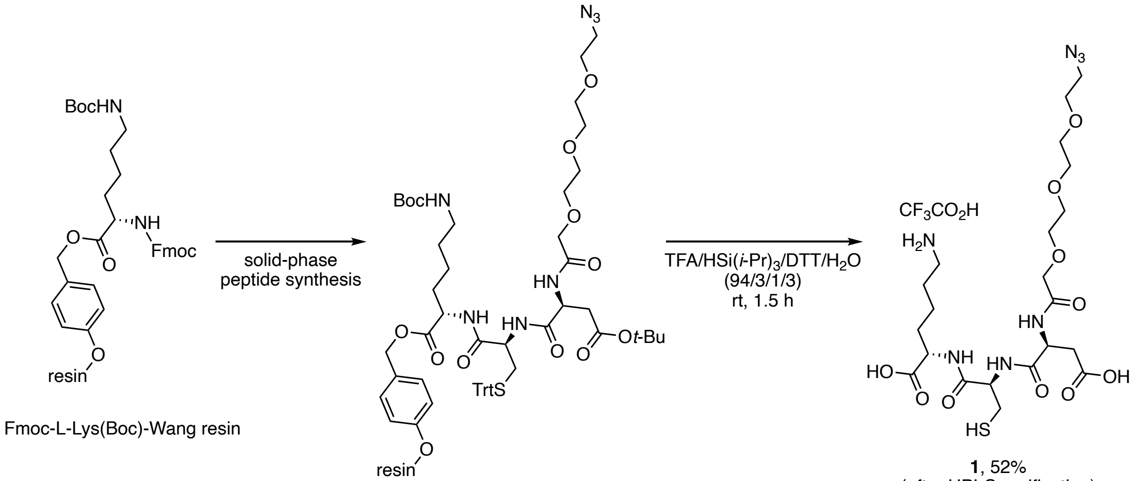

1, 52%
(after HPLC purification)

Fmoc-L-Lys(Boc)-Wang resin (100–200 mesh, 0.56 mmol/g for Lys, 893 mg, 0.50 mmol for Lys) and DMF (5.0 mL) were added to a column (diameter, 2 cm) equipped to a solid-phase peptide synthesizer (Kokusan Chemical, KMS-3). The suspension was stirred at room temperature for 1 h. The solution was discarded, and a mixture of DMF/piperidine (6.0 mL/1.5 mL) was added to the column. The suspension was stirred for 10 min at room temperature. The solution was discarded, and the resin was washed with DMF (2 mL × 10). A solution of Fmoc-L-Cys(trt)-OH (879 mg, 1.50 mmol, 3 equiv), HATU (570 mg, 1.50 mmol, 3 equiv), HOAt (204 mg, 1.50 mol, 3 equiv), and EtN(*i*-Pr)$_2$ (523 mL, 3.00 mmol, 6 equiv) in DMF (7.0 mL) was added to the resin. The suspension was stirred for 1.5 h at room temperature. The solution was discarded, and the resin was washed with DMF (2 mL × 10). A mixture of DMF/piperidine (6.0 mL/1.5 mL) was added to the resin, and the suspension was stirred for 10 min at room temperature. The solution was discarded, and the resin was washed with DMF (2 mL × 10). A solution of Fmoc-L-Asp(t-Bu)-OH (626 mg, 1.50 mmol, 3 equiv), HATU (570 mg, 1.50 mmol, 3 equiv), HOAt (204 mg, 1.50 mol, 3 equiv),and EtN(*i*-Pr)$_2$ (523 mL, 3.00 mmol, 6 equiv) in DMF (7.0 mL) was added to the resin. The suspension was stirred for 1.5 h at room temperature. The solution was discarded, and the resin was washed with DMF (2 mL × 10). A solution of 2-(2-(2-(2-azidoethoxy)ethoxy)ethoxy)acetic acid (350 mg, 1.50 mmol, 3 equiv), HATU (570 mg, 1.50 mmol, 3 equiv), HOAt (204 mg, 1.50 mol, 3 equiv), and EtN(*i*-Pr)$_2$ (523 mL, 3.00 mmol, 6 equiv) in DMF (7.0 mL) was added to the resin. The suspension was stirred for 1.5 h at room temperature. The solution was discarded, and the resin was washed with DMF (2 mL × 5) and CH_2Cl_2 (2 mL × 5). A mixture of trifluoroacetic acid (9.4 mL), water (0.30 mL), HSi(*i*-Pr)$_3$ (0.10 mL), and DTT

(300 mg) was added to the resin. The suspension was stirred at room temperature for 1.5 h. After the reaction, the suspension was filtered, and the filtrate was collected in a 50 mL tube while washing the resin with trifluoroacetic acid (2 mL × 5). The combined solution was concentrated to ca. 1 mL by purging argon gas into the solution. Diethyl ether (40 mL) was added to the residue. The mixture was allowed to stand at –20 °C for 30 min. The resulting colorless precipitate was collected by centrifugation. The colorless solid was decantated with diethyl ether (20 mL × 2). The colorless solid was purified by HPLC (column, COSMOSIL 5C18-AR-II, 20 × 250 mm; eluent, H_2O/MeCN + 0.1 v/v% trifluoroacetic acid = 95/5 → 55/45; flow rate, 10 mL/min).

Yield 181 mg (0.261 mmol, 52%); colorless solid; mp 158 °C (decomposed); ^{1}H NMR (400 MHz, $CDCl_3$) δ 4.43 (dd, J = 6.8, 6.0 Hz, 1H), 4.29 (dd, J = 9.6, 5.2 Hz, 1H), 4.09–4.00 (m, 2H), 3.67–3.61 (m, 11H), 3.40 (t, J = 4.8 Hz, 2H), 2.93–2.76 (m, 6H), 1.87–1.80 (m, 1H), 1.73–1.67 (m, 1H), 1.63–1.55 (m, 2H), 1.41–1.29 (m, 2H); ^{13}C NMR (100 MHz, $CDCl_3$) δ 175.1, 174.1, 172.8, 172.1, 171.6, 163.0 (d, $^1J_{C\text{-}F}$ = 35.5 Hz), 116.3 (q, $^2J_{C\text{-}F}$ = 293.2 Hz), 70.4, 69,7, 69.5, 69.4, 69.2, 55.6, 52.5, 50.2, 49.7, 39.2, 35.3, 29.8, 26.1, 25.2, 22.0; ^{19}F NMR ($CDCl_3$) δ –75.5; IR (ZnSe) 2932, 2016, 1714, 1643, 1518, 1180, 1134, 721 cm^{-1}; HRMS (ESI$^+$, m/z) [M – $CF_3CO_2^-$]$^+$ calcd for $C_{21}H_{38}N_7O_{10}S^+$, 580.2395; found 580.2396.

Pyropheophorbide a *N*-hydroxysuccinimidyl ester (**4**)

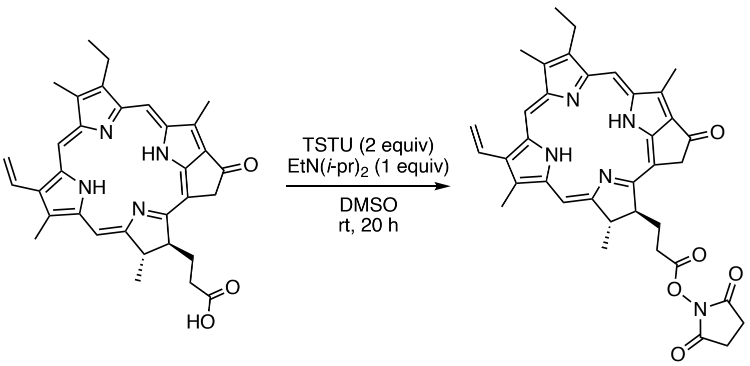

4, 76%

To a solution of pyropheophorbide a (10.7 mg, 10.0 μmol, 1 equiv) in DMSO (0.50 mL) in a 4 mL vial was added EtN(*i*-Pr)$_2$ (3.5 μL, 20 μmol, 1 equiv) followed by TSTU (12.0 mg, 39.9 μmol, 2 equiv). The reaction mixture was stirred at room temperature for 20 h under argon atmosphere. After the reaction, the solution was naturized by formic acid (0.8 μL, 21 μmol, 1 equiv). The solution was purified by HPLC (column, COSMOSIL 5C18-AR-II; 10 × 250 mm; eluent, H_2O/MeCN + 0.1 v/v% formic acid = 1/1 to 0/1, flow rate 5 mL/min).

Yield 9.6 mg (15 μmol, 76%); green solid; mp 212 °C (decomposed); ^{1}H NMR (400 MHz, $CDCl_3$): δ 9.37 (s, 1H), 9.26 (s, 1H), 8.49 (s, 1H), 7.88 (dd, J = 17.6, 11.6 Hz, 1H), 5.16–5.02 (m,

2H), 4.45–4.39 (m, 1H), 4.34–4.32 (m, 1H), 3.59–3.53 (m, 5H), 3.31 (s, 3H), 3.11 (s, 3H), 2.85–2.68 (m, 5H), 2.57–2.51 (m, 1H), 2.24–2.15 (m, 1H), 1.73 (d, *J* = 7.6 Hz, 3H), 1.58 (t, 3H), 0.31 (s, 1H), –1.85 (s, 1H); ^{13}C NMR (100 MHz, $CDCl_3$): δ 196.4, 171.3, 169.2, 168.5, 160.0, 154.9, 149.2, 145.1, 141.8, 138.0, 136.4, 136.1, 131.9, 130.8, 129.3, 122.8, 106.4, 104.3, 97.4, 93.4, 51.2, 50.0, 48.1, 29.8, 28.4, 25.8, 23.3, 19.6, 17.6, 12.31, 12.28, 11.4; IR (ZnSe) 2866, 1738, 1683, 1616, 1499, 1347, 1204, 981 cm^{-1}; HRMS (ESI, m/z): [M + H$^+$]$^+$ calcd for $C_{37}H_{38}N_5O_5^+$, 632.2867; found 632.2870. The spectral data was in accordance with the literature.[S4]

(*Z*)-2-Methoxy-4-(3,4,5-trimethoxystyryl)phenyl 6-(4-(*tert*-butoxy)-4-oxobutoxy)-2-methoxyindolizine-3-carboxylate (**S1**)

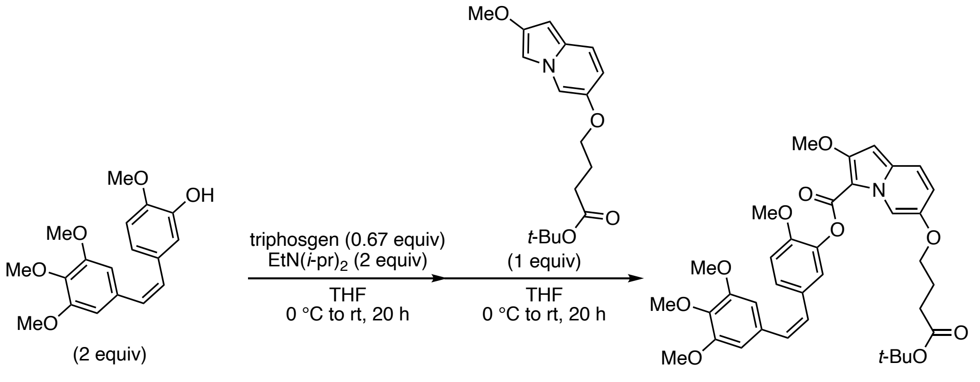

S1, 36%

To a solution of triphosgene (496 mg, 0.167 mmol, 0.667 equiv) in THF (0.50 mL) in a 4 mL vial at 0 °C was added a solution of combretastatin A4 (158 mg, 0.499 mmol, 2 equiv) in THF (1.0 mL), followed by a solution of EtN(*i*-Pr)$_2$ (87.1 mL, 0.500 mmol, 2 equiv) in THF (0.50 mL). The reaction mixture was stirred at room temperature for 20 h under argon atmosphere. After the reaction, the reaction mixture was diluted with EtOAc (15 mL) and washed with water (15 mL × 3). The organic layer was dried over sodium sulfate, filtered, and concentrated. The residue was dissolved in THF (1.0 mL) and added to a 4 mL vial. The solution was cooled to 0 °C and a solution of *tert*-butyl 4-((2-methoxyindolizin-6-yl)oxy)butanoate (72.3 mg, 0.250 mmol, 1 equiv) in THF (1.0 mL) was added. The reaction mixture was stirred at room temperature for 20 h under argon atmosphere. After the reaction, the reaction mixture was diluted with EtOAc (15 mL) and washed with water (15 mL × 3). The organic layer was dried over sodium sulfate, filtered, and concentrated. The residue was purified by silica gel flash column chromatography (hexane/EtOAc = 1/0 to 1/1). The compound was further purified by GPC column chromatography.

Yield 57.7 mg (89.1 mmol, 35.7%); colorless solid; mp 53.0–56.4 °C; TLC R_f = 0.51 (hexane/EtOAc = 2/1); ^{1}H NMR (400 MHz, $CDCl_3$) δ 9.13 (d, *J* = 2.0 Hz, 1H), 7.24 (m, 2H), 7.17 (dd, *J* = 8.4, 2.0 Hz, 1H), 6.91–6.87 (m, 2H), 6.59 (s, 2H), 6.49–6.40 (m, 2H), 6.01 (s, 1H), 3.97–3.94 (m, 5H), 3.82 (s, 3H), 3.81 (s, 3H), 3.75 (s, 6H), 2.40 (t, *J* = 8.0 Hz, 2H), 2.09–2.02 (m,

2H), 1.44 (s, 9H); ^{13}C NMR (100 MHz, $CDCl_3$) δ 172.6, 159.8, 157.9, 153.1, 151.3, 148.4, 139.6, 137.3, 133.6, 132.8, 130.1, 129.1, 129.0, 127.6, 124.4, 118.7, 117.4, 112.2, 111.8, 106.1, 101.7, 85.2, 80.6, 68.1, 61.1, 58.3, 56.3, 56.2, 32.2, 28.3, 24.9; IR (ZnSe) 2936, 1727, 1680, 1580, 1504, 1454, 1418, 1267, 1240, 1126, 1090, 1009, 808 cm^{-1}; HRMS (ESI, m/z): [M + H$^+$]$^+$ calcd for $C_{36}H_{42}NO_{10}^+$, 648.2803; found 648.2808.

(*Z*)-2-Methoxy-4-(3,4,5-trimethoxystyryl)phenyl 6-(4-((2,5-dioxopyrrolidin-1-yl)oxy)-4-oxobutoxy)-2-methoxyindolizine-3-carboxylate (**5**)

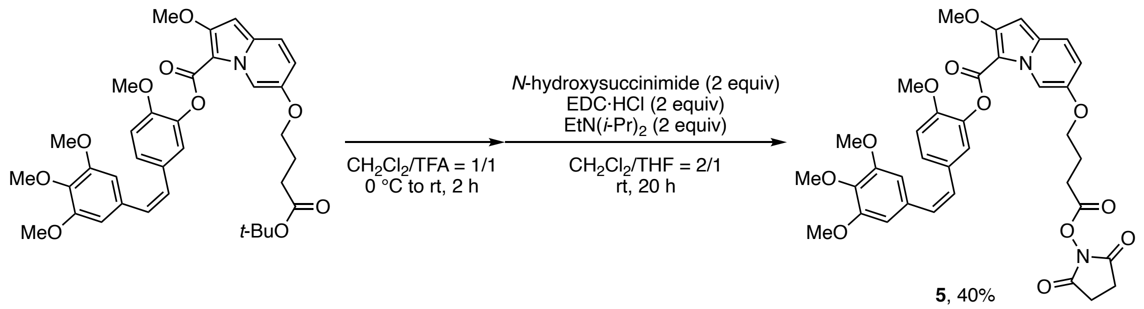

To a solution of **S1** (32.4 mg, 49.9 mmol, 1 equiv) in CH_2Cl_2 (1.0 mL) in a 20 mL vial at 0 °C was added trifluoroacetic acid (1.0 mL). The mixture was allowed to warm to room temperature and stirred for 2 h under argon atmosphere. After the reaction, the reaction mixture was evaporated, and the residue was dried under vacuum. The residue was dissolved in a mixture of CH_2Cl_2 (0.40 mL) and THF (0.20 mL), and to this solution were added *N*-hydroxysuccinimide (11.5 mg, 99.9 mmol, 2 equiv), EtN(*i*-Pr)$_2$ (17.4 mL, 99.9 mmol, 2 equiv), and 1-(3-dimethylaminopropyl)-3-ethylcarbodiimide hydrochloride (19.2 mg, 100 mmol, 2 equiv). The reaction mixture was stirred at room temperature for 20 h under argon atmosphere. After the reaction, the reaction mixture was diluted with EtOAc (15 mL) and washed with water (15 mL × 3). The organic layer was dried over sodium sulfate, filtered, and the filtrate was concentrated. The residue was purified by silica gel flash chromatography (hexane/EtOAc = 1/0 to 0/1). The compound was further purified by GPC column chromatography.

Yield 13.6 mg (19.8 mmol, 39.6%); colorless solid; mp 71.5–74.7 °C; TLC R_f = 0.45 (hexane/EtOAc = 1/1); ^{1}H NMR (400 MHz, $CDCl_3$) δ 9.14 (d, *J* = 2.0 Hz, 1H), 7.28–7.23 (m, 2H), 7.16 (d, *J* = 8.8, 2.0 Hz, 1H), 6.93 (dd, *J* = 9.6, 2.4 Hz, 1H), 6.88 (d, *J* = 8.8 Hz, 1H), 6.59 (s, 2H), 6.49–6.40 (m, 2H), 6.02 (s, 1H), 4.04 (t, *J* = 6.0 Hz, 2H), 3.95 (s, 3H), 3.82 (s, 3H), 3.81 (s, 3H), 3.74 (s, 6H), 2.86–2.82 (m, 6H), 2.25–2.18 (m, 2H); ^{13}C NMR (100 MHz, $CDCl_3$) δ 169.3, 168.4, 159.7, 157.9, 153.1, 151.3, 148.2, 139.6, 137.3, 133.7, 132.8, 130.1, 129.2, 129.0, 127.6, 124.3, 118.7, 117.5, 112.2, 119.0, 106.1, 103.5, 101.7, 85.3, 67.2, 61.0, 58.3, 56.3, 56.2, 28.0, 25.8, 24.5; IR (ZnSe) 2938, 1738, 1674, 1504, 1454, 1418, 1350, 1267, 1206, 1088, 1007, 806 cm^{-1}; HRMS (ESI, m/z): [M + H$^+$]$^+$ calcd for $C_{36}H_{37}N_2O_{12}^+$, 689.2341; found 689.2346.

13. Synthetic procedures for gold nanoclusters

Synthesis of **AuNc1**

In a 50 mL round-bottom flask, a solution of tripeptide thiol **1** (139 mg, 200 μmol, 1 equiv) in 6.0 mL of ultrapure water was mixed with a solution of $HAuCl_4 \cdot 4H_2O$ (41.2 mg, 100 μmol, 0.5 equiv) in 6 mL of ultrapure water. The solution was stirred at room temperature for 30 min under an argon atmosphere. To this solution, a solution of phenol (565 mg, 6.00 mmol, 30 equiv) and NaOH (240 mg, 6.00 mmol, 30 equiv) in 6.0 mL of ultrapure water was added. The mixture was stirred at 50 °C for 48 h under an argon atmosphere. After the reaction, the solution was neutralized by adding formic acid (230 μL, 6.00 mmol, 30 equiv). The solution was subjected to ultrafiltration using an Amicon Ultra filter unit (Nominal Molecular Weight Limit (NMWL) 10000) for 5 cycles. The concentrated solution was freeze-dried, yielding a black solid (29.1 mg).

Au determined by ICP-AES, 66.3 wt%. Yield based on Au, 97.9%.

Synthesis of **AuNc2**

In a 20 mL vial, a solution of $CuSO_4 \cdot 5H_2O$ (0.499 mg/50 μL, 50 μL, 2.00 μmol) in ultrapure water was mixed with a solution of tris(3-hydroxypropyltriazolylmethyl)amine (THPTA, 4.35 mg/250 μL, 250 μL, 10.0 μmol) in ultrapure water, followed by a solution of sodium L-ascorbate (0.792 mg/100 μL, 100 μL, 4.00 μmol) in ultrapure water. The resulting solution of copper(I) catalyst was added to a solution of **AuNc1** (3.2 mg), *N*-propargyltrifluoroacetamide (**2**, 1.21 mg, 8.01 μmol), and aminoguanidine hydrochloride (0.884 mg, 8.00 μmol) in 1.1 mL of sodium phosphate buffer (pH 7.4, 0.1 M). The mixture was stirred at room temperature for 20 h under an argon atmosphere. After the reaction, the solution was diluted with a solution of ethylenediaminetetraacetic acid (EDTA) in ultrapure water (50 mM, pH 8.0, 10 mL). The solution was subjected to ultrafiltration using an Amicon Ultra filter unit (NMWL 10000) for 5 cycles. The concentrated solution was freeze-dried, yielding a black solid (2.9 mg).

Au determined by ICP-AES, 62 wt%. Yield based on Au, 84%

Synthesis of **AuNc3**

In a 20 mL vial, a solution of $CuSO_4 \cdot 5H_2O$ (1.4 mg, 5.6 μmol) in 150 μL of ultrapure water was mixed with a solution of THPTA (12.4 mg, 28.5 μmol) in 750 μL of ultrapure water, followed by a solution of sodium L-ascorbate (2.3 mg, 12 μmol) in 300 μL of ultrapure water. The resulting solution of copper (I) catalyst was added to a solution of **AuNc1** (9.6 mg), cyclic RGD peptide **3** (18.3 mg, 22.9 μmol), and aminoguanidine hydrochloride (2.5 mg, 23 μmol) in 3.3 mL of sodium phosphate buffer (pH 7.4, 0.1 M). The mixture was stirred at room temperature for 20 h under an argon atmosphere. After the reaction, the solution was diluted with a solution of

ethylenediaminetetraacetic acid (EDTA) in ultrapure water (50 mM, pH 8.0, 10 mL). The solution was subjected to ultrafiltration using an Amicon Ultra filter unit (NMWL 10000) for 5 cycles. The concentrated solution was freeze-dried, yielding a black solid (10.6 mg).
Au determined by ICP-AES, 52 wt%. Yield based on Au, 86%.

Synthesis of **AuNc4**
In a 4 mL vial, **AuNc3** (3.0 mg) was mixed with a solution of $NaHCO_3$ in ultrapure water (100 mM, 1.4 mL, 1.4 mmol), followed by a solution of pyropheophorbide a derivative **4** in DMSO (2.86 mM, 700 μL, 2.00 μmol). The solution was stirred at room temperature for 20 h under an argon atmosphere. After the reaction, the mixture was diluted with DMSO (3.5 mL) and ultrapure water (10.5 mL). The solution was then subjected to ultrafiltration using an Amicon Ultra filter unit (NMWL 10000) for 5 cycles. The concentrated solution was freeze-dried, yielding a green solid (2.4 mg).
Au determined by ICP-AES, 42 wt%. Yield based on Au, 65%.

Synthesis of **AuNc5**
In a 4 mL vial, **AuNc3** (3.0 mg) was mixed with a solution of $NaHCO_3$ in ultrapure water (100 mM, 1.4 mL, 1.4 mmol), followed by a solution of indolizine derivative **5** in DMSO (1.43 mM, 700 μL, 1.00 μmol). The solution was stirred at room temperature for 6 h under an argon atmosphere. Next, a solution of pyropheophorbide a derivative **4** in DMSO (2.86 mM, 700 μL, 2.00 μmol) was added to the reaction mixture. The solution was stirred at room temperature for an additional 20 h under an argon atmosphere. After the reaction, the mixture was diluted with DMSO (3.5 mL) and ultrapure water (10.5 mL). The solution was then subjected to ultrafiltration using an Amicon Ultra filter unit (NMWL 10000) for 5 cycles. The concentrated solution was freeze-dried, yielding a green solid (3.0 mg).
Au determined by ICP-AES, 41 wt%. Yield based on Au, 79%.

Synthesis of **AuNc6**
In a 4 mL vial, **AuNc3** (3.0 mg) was mixed with a solution of $NaHCO_3$ in ultrapure water (100 mM, 1.4 mL, 1.4 mmol), followed by a solution of indolizine derivative **5** in DMSO (2.86 mM, 1.4 mL, 4.00 μmol). The solution was stirred at room temperature for 20 h under an argon atmosphere. After the reaction, the mixture was diluted with DMSO (3.5 mL) and ultrapure water (10.5 mL). The solution was then subjected to ultrafiltration using an Amicon Ultra filter unit (NMWL 10000) for 5 cycles. The concentrated solution was freeze-dried, yielding a black solid (3.1 mg).
Au determined by ICP-AES, 40 wt%. Yield based on Au, 79%.

Synthesis of **AuNcS1**

In a 4 mL vial, a solution of **AuNc1** (1.6 mg) in 300 μL of sodium phosphate buffer (pH 7.4, 0.1 M) was mixed with a solution of a cyanine dye **S1** (2.0 mg, 2.0 μmol) in 300 μL of ultrapure water. The mixture was stirred at room temperature for 20 h under an argon atmosphere. After the reaction, the reaction mixture was diluted with 14 mL of ultrapure water. The solution was subjected to ultrafiltration using an Amicon Ultra filter unit (NMWL 10000) for 5 cycles. The concentrated solution was freeze-dried, yielding a blue solid (1.8 mg).

Au determined by ICP-AES, 42 wt%. Yield based on Au, 71%.

14. Procedure for TEM measurements

Transmission electron microscopy (TEM) images of the gold nanoclusters were obtained using a field emission transmission electron microscope (JEOL, JEM-2100F). The measurement sample was prepared by placing a solution of the gold nanoclusters (0.05 mg/mL) in ethanol (95%) on a carbon-supported copper grid (STEM, HRC-C10) and drying it under vacuum.

15. Procedure for ICP-AES measurements

Inductively coupled plasma atomic emission spectroscopy (ICP-AES) measurements were performed on an ICPS-8100 sequential plasma spectrometer (Shimazu Co.). Calibration curves were prepared in the range of 0–20 ppm using gold standard solutions.

The sample solutions were prepared according to the following procedures. To a solution of gold nanoclusters in ultrapure water (1.0 mg/mL, 100 μL) in a 20 mL vial was added nitric acid (69 wt%, 250 μL) followed by hydrochloric acid (35 wt%, 750 μL). The solution was diluted with ultrapure water (3.9 mL). Thus, the final concentration of the gold nanoclusters was 20 ppm.

Table S2. Gold contents of gold nanoclusters (wt%).

**AuNc1**	66.3
**AuNc2**	62
**AuNc3**	52
**AuNc4**	42
**AuNc5**	41
**AuNc6**	40
**AuNcS1**	42

16. Procedure for MTT assay

A549 and WI-38 cells were purchased from RIKEN Cell Bank. The cells were used after being subcultured at least three times. A549 in DMEM or WI-38 cells in MEM supplemented with 10% fetal bovine serum albumin were seeded into each well of 96-well plates (ca. 5×10^4 cells/well for A549 cells; ca. 1×10^5 cells/well for WI-38 cells) and incubated at 37 °C for 24 h (for A549 cells) or 48 h (for WI-38 cells) under a humidified CO_2 (5%) atmosphere. The adhesion of the cells to the 96-well plates was confirmed by optical microscopy. The culture medium in the well was discarded, and the medium (100 μL) containing **AuNc4–6** (0–20 μg/mL) was added, followed by incubation for 1 h at 37 °C under a humidified CO_2 (5%) atmosphere. The medium containing the samples was then removed and replaced with fresh the medium (100 μL) without samples. The 96-well plate containing the cells was photoirradiated with red LED (Kessil H160 Tuna Flora LED lamp, red channel, 660 nm) for 5 min. The optical path length from the light source to the 96-well plate was 5 cm. After the photoirradiation, the cells were incubated for 20 h at 37 °C under a humidified CO_2 (5%) atmosphere. The cell viability was evaluated using the MTT cell proliferation assay kit (Cayman Chemical).

17. Procedure for confocal fluorescence microscopy imaging

To a 22 mm circular microscope cover glass placed on a cell culture dish (35 mm), A549 or WI-38 cells (ca. 10^5 cells) suspended in D-10 medium (1 mL) were seeded and incubated at 37 °C for 24 h under humidified CO_2 atmosphere (5%). The medium was replaced with fresh D-10 medium containing gold nanoclusters **AuNc4** or **AuNc5** (4 μg/mL, 1 mL). The cells were incubated at 37 °C for 1 h under humidified CO_2 atmosphere (5%). After the cells were washed with PBS(–), the cover glass was taken out from the culture and mounted on the stage of a fluorescence microscope with a microscope glass slide. Fluorescence images were taken using a Nikon A1 MP multi-photon confocal laser scanning microscope. The excitation and fluorescence wavelength were 638 nm and 688 nm, respectively.

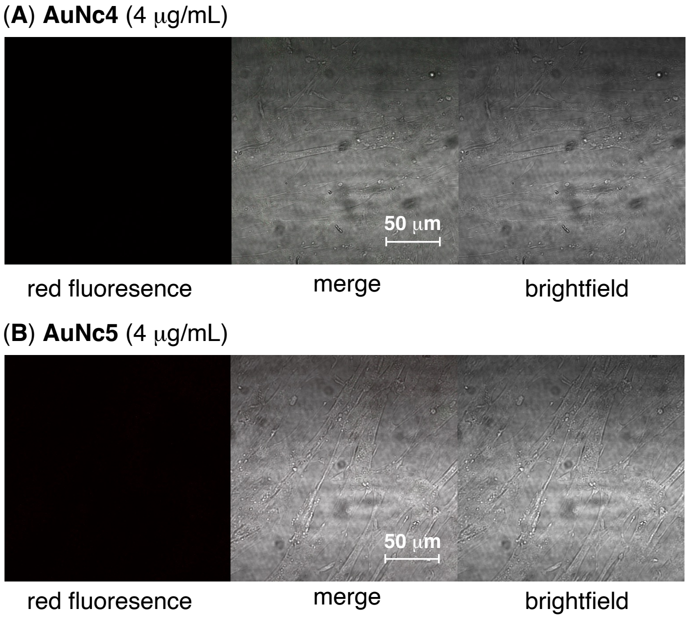

Fig. S8 Confocal fluorescence microscopy imaging of WI-38 cells incubated with **AuNc4** (A, 4 μg/mL) or **AuNc5** (B, 4 μg/mL) for 1 h in D-10 medium. The excitation and fluorescence wavelength were 638 nm and 688 nm, respectively. Almost no fluorescence of **AuNc4** and **AuNc5** were observed, indicating that they were barely taken up by WI-38 cells.

18. References

S1 Jin, R.; Higaki, T. *Commun. Chem.* **2021**, *4*, 28.

S2 Neirynck, P.; Brinkmann, J.; An, Q.; van der Schaft, D. W. J.; Milroy, L.-G.; Jonkheijm, P.; Brunsveld, L. Supramolecular control of cell adhesion viaferrocene–cucurbit[7]uril host–guest binding on gold surfaces. *Chem. Commun.* **2013**, *49*, 3679–3681.

S3 Watanabe, K.; Kuratsu, A.; Hashizume, D.; Niwa, T.; Hosoya, T. Red light-induced conjugation of amines through amide bond formation triggered via photooxidation of 3-acylindolizines. *Commun. Chem.* **2022**, *5*, 91.

S4 Helmreich, M.; Ermilov, E. A.; Meyer, M.; Jux, N.; Hirsch, A.; Röder, B. Dissipation of electronic excitation energy within a C_{60} [6:0]-hexaadduct carrying 12 pyropheophorbide a moieties. *J. Am. Chem. Soc.* **2005**, *127*, 8376–8385.

19. NMR charts

^1H, ^{13}C, and ^{19}F NMR charts of **1** in D_2O

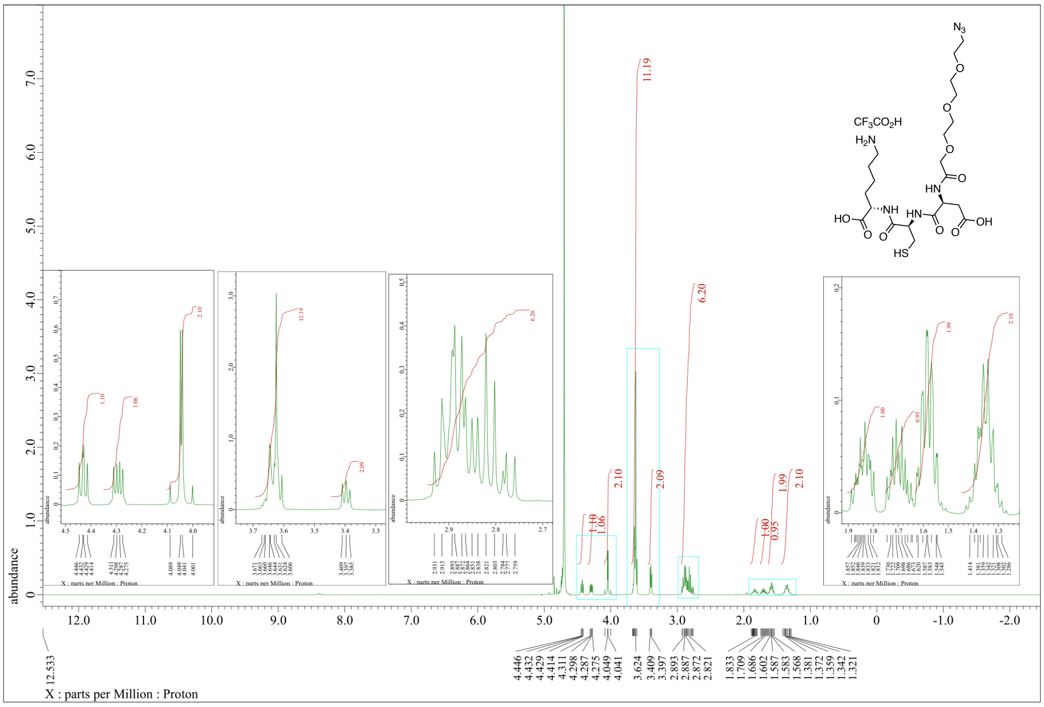

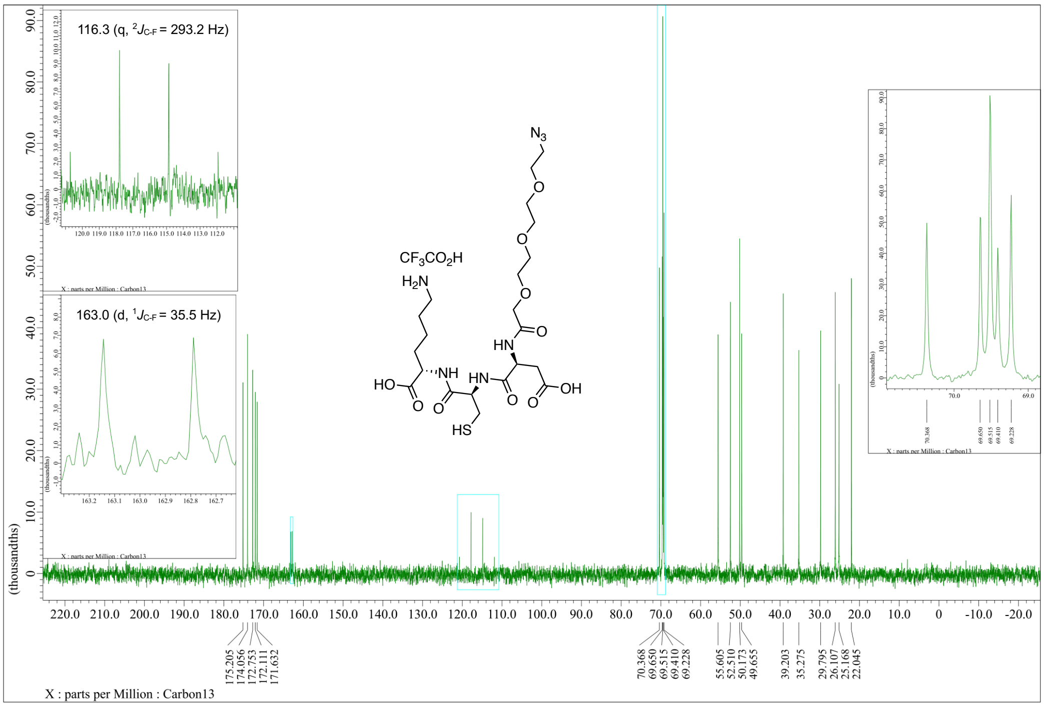

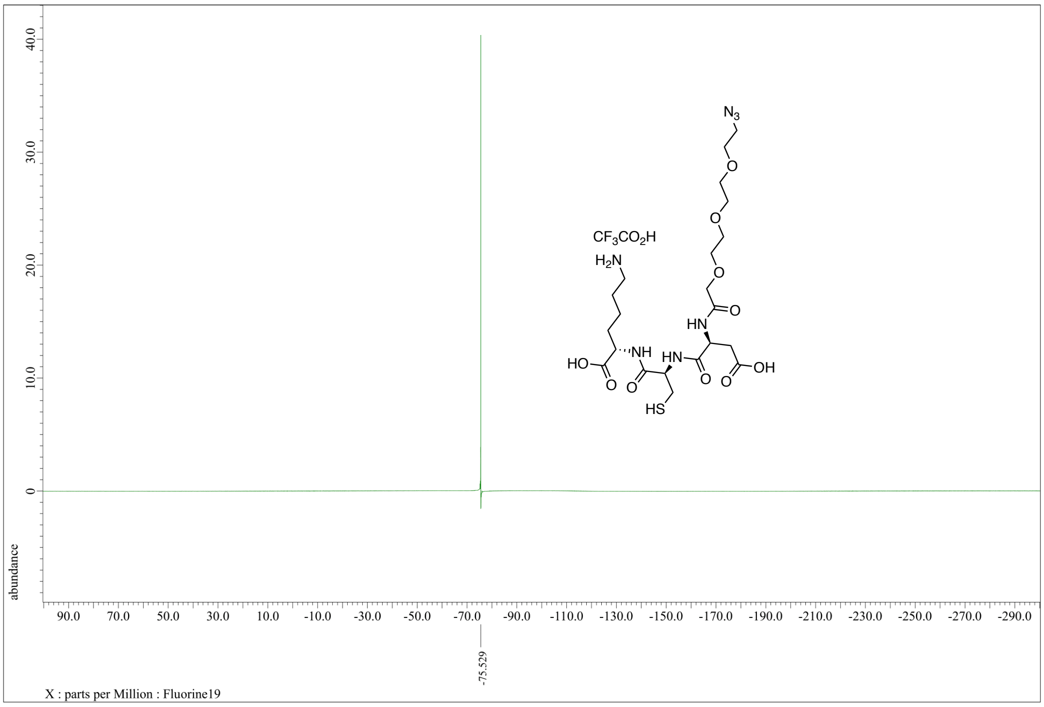
CF3CO2H
H2N
NH
HO
O
O
HN
HS
HN
O
O
OH
O
O
O
N3
abundance
0
10.0
20.0
30.0
40.0
90.0 70.0 50.0 30.0 10.0 -10.0 -30.0 -50.0 -70.0 -90.0 -110.0 -130.0 -150.0 -170.0 -190.0 -210.0 -230.0 -250.0 -270.0 -290.0
-75.529
X : parts per Million : Fluorine19

^{1}H and ^{13}C NMR charts of **4** in $CDCl_3$

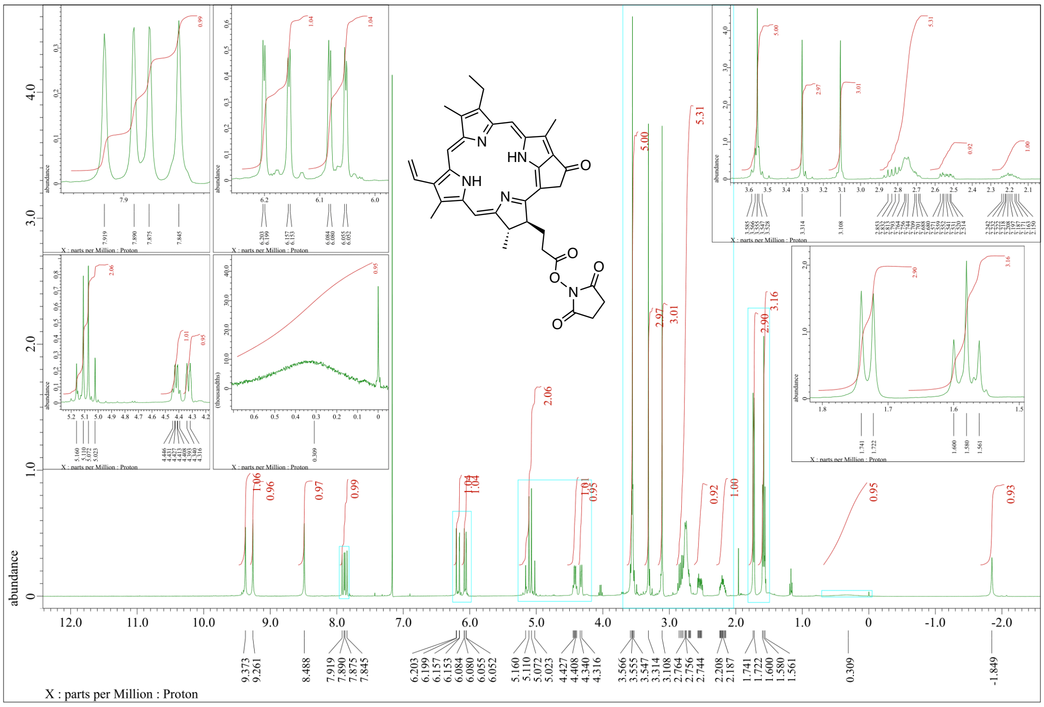

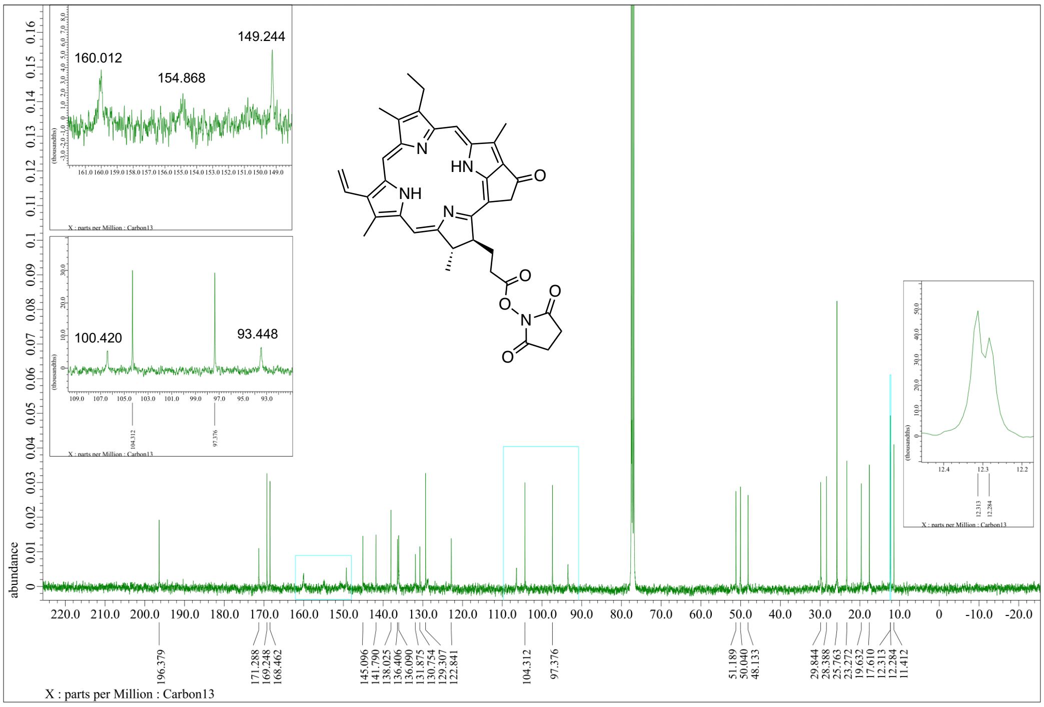

^{1}H and ^{13}C NMR charts of **S1** in $CDCl_3$

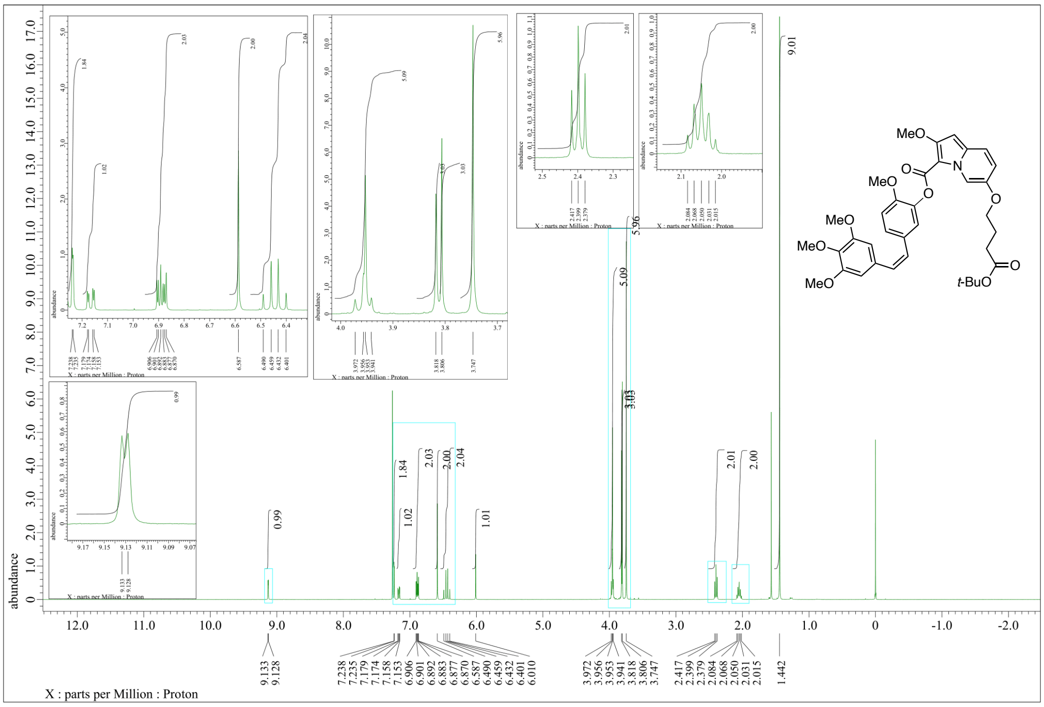

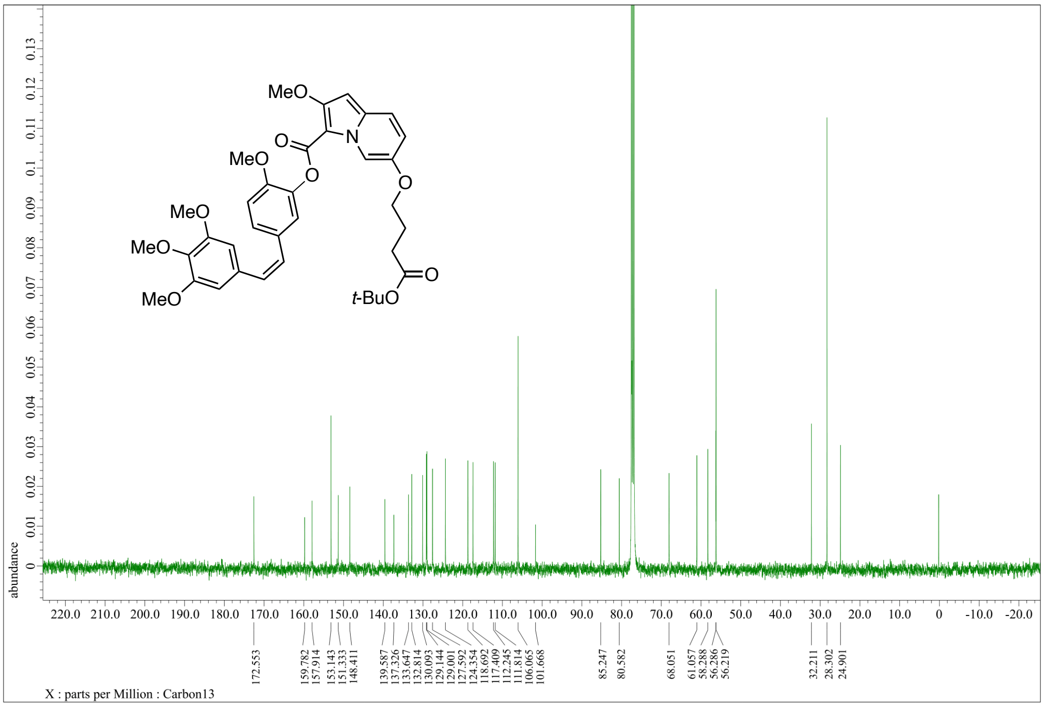

^{1}H and ^{13}C NMR charts of **5** in $CDCl_3$

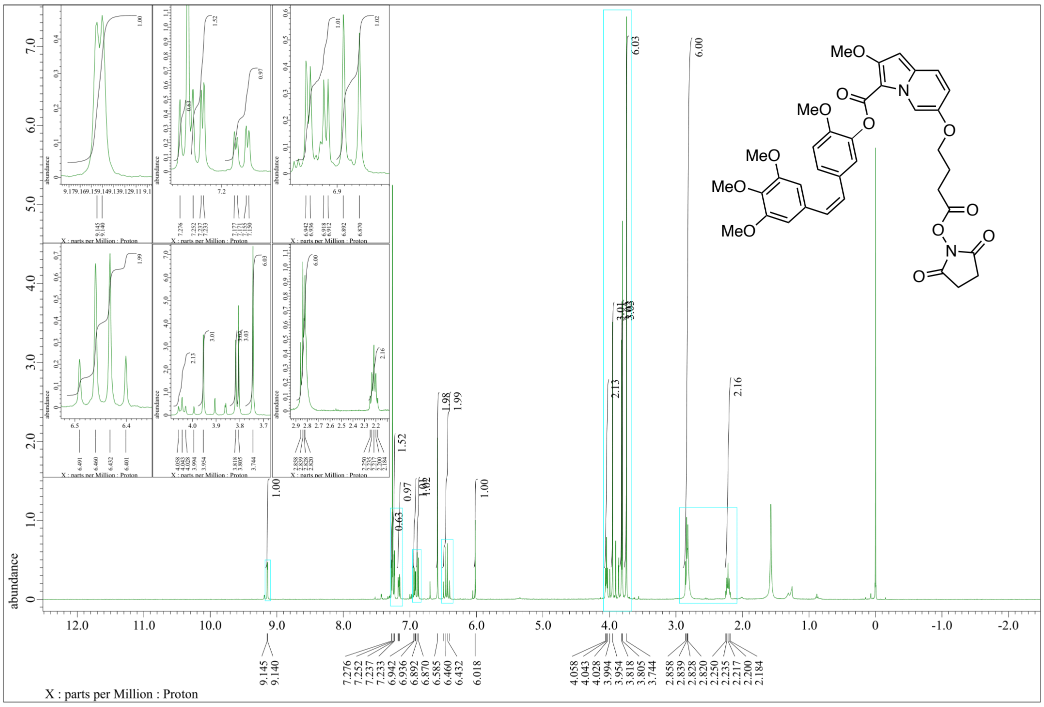

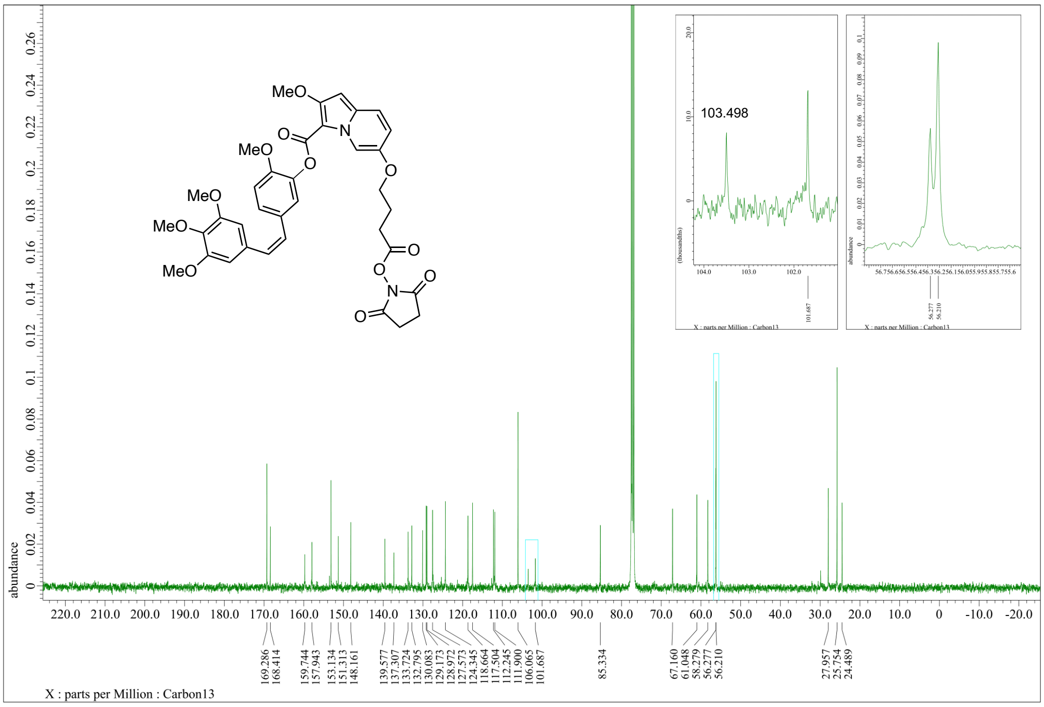

Supplement: SC-015-D3SC04365G-s001 [file SC-015-D3SC04365G-s001.pdf]
